# Direct Photopatterning of Green Solvent‐Processed 2D Nanomaterials for Wafer‐Scale Electronics

**DOI:** 10.1002/adma.202505917

**Published:** 2025-07-21

**Authors:** In Cheol Kwak, Se‐Jin Kim, Wan Ho Cho, Jihyun Kim, Seonkwon Kim, Yonghyun Albert Kwon, Vlastimil Mazánek, Zdeněk Sofer, Jinho Keum, Yuchan Heo, Moon Sung Kang, BongSoo Kim, Joohoon Kang, Jeong Ho Cho

**Affiliations:** ^1^ Department of Chemical and Biomolecular Engineering Yonsei University Seoul 03722 Republic of Korea; ^2^ School of Advanced Materials Science and Engineering Sungkyunkwan University (SKKU) Suwon 16419 Republic of Korea; ^3^ Department of Chemistry Ulsan National Institute of Science and Technology (UNIST) Ulsan 44919 Republic of Korea; ^4^ Department of Inorganic Chemistry University of Chemistry and Technology Prague Technická 5, Prague 6 Prague 166 28 Czech Republic; ^5^ Department of Chemical and Biomolecular Engineering Sogang University Seoul 04107 Republic of Korea; ^6^ Institute of Emergent Materials Ricci Institute of Basic Science Sogang University Seoul 04107 Republic of Korea; ^7^ Graduate School of Semiconductor Materials and Device Engineering Ulsan National Institute of Science and Technology (UNIST) 50 UNIST‐gil Ulsan 44919 Republic of Korea; ^8^ Graduate School of Carbon Neutrality Ulsan National Institute of Science and Technology (UNIST) 50 UNIST‐gil Ulsan 44919 Republic of Korea

**Keywords:** 2D nanomaterials, green solvents, hansen solubility, photopatterning, transistors

## Abstract

Solution‐processed 2D nanomaterials have emerged as key building blocks for the large‐scale assembly of functional electronic devices. Solution processing enables the formation of electronically active percolated networks by leveraging van der Waals (vdW) interactions between individual 2D nanosheets. While effective vdW interactions are expected to minimize potential energy barriers and contact resistances between nanosheets, undesired residues from material synthesis or device fabrication processes may remain at the interface. In particular, the ideal solvent candidates for optimizing the stability of 2D dispersions are typically difficult to remove due to their high boiling points and exhibit environmental toxicity. Additionally, conventional patterning processes require multiple solvents, which can disrupt vdW interfaces and degrade device performance. To address these challenges, a comprehensive process that combines 2D dispersion preparation with a cross‐linker‐based direct photopatterning technique is developed using an eco‐friendly green solvent. To enable this process, the stability of 2D nanomaterials and ultraviolet light‐sensitive cross‐linkers is thoroughly analyzed using Hansen solubility parameters. The developed process successfully enables the preparation of stable dispersions of cross‐linkers and 2D nanomaterials, including graphene, molybdenum disulfide, tungsten diselenide, and hafnium disulfide, which can then be assembled via vdW interactions to create large‐scale functional electronic devices.

## Graphical abstract

A photocross‐linking strategy is proposed for the patterning and integration of scalable vertical van der Waals heterostructures such as field‐effect transistors, logic gates, and memory devices composed of 2D nanomaterials by UV light‐sensitive cross‐linker in isopropyl alcohol. To optimize the alcohol‐based dispersion system, Hansen solubility parameter analysis is performed to identify a solvent capable of simultaneously stabilizing both the 2D nanomaterials and the cross‐linker.

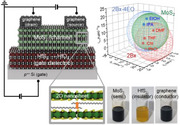

## 1 Introduction

Solution‐based processing of 2D nanomaterials has been extensively explored as a promising route for producing diverse electronic‐grade 2D nanosheets in a scalable manner.^[^ ^1^, ^2^, ^3^, ^4^, ^5^, ^6^, ^7^ ^]^ Owing to their unique structural properties, such as a high aspect ratio, dangling‐bond‐free surfaces, and atomically clean surfaces, individual 2D nanosheets can be assembled into electronically active mosaic‐like thin films by partially overlapping of nanosheets with van der Waals (vdW) interfaces, enabling scalable electronic applications.^[^ ^8^, ^9^, ^10^, ^11^, ^12^ ^]^ The percolated networks of 2D nanosheets with diverse electronic properties are usually vertically stacked to create functional electronic devices at the wafer scale.^[^ ^13^, ^14^, ^15^ ^]^ Although this approach is considered a promising approach for the practical demonstration of 2D nanomaterial‐based electronics, the realization of 2D nanomaterials as electronic components for vertically assembled vdW heterostructures remains a significant challenge owing to the presence of residual solvents from the processes, the lack of a precise patterning process, and the instability of multi‐stacking integration.^[^ ^16^, ^17^, ^18^, ^19^, ^20^, ^21^ ^]^

The previously reported research on solution‐based processing of 2D nanomaterials has revealed that 2D nanomaterials exhibit remarkable stability in organic solvents with high boiling points because of their similar surface energy (e.g., N‐methyl pyrrolidone (NMP) and N,N‐dimethylformamide (DMF)).^[^ ^7^, ^22^, ^23^, ^24^, ^25^ ^]^ However, such organic solvents have a detrimental impact on 2D nanomaterial‐based electronic applications because they are difficult to remove owing to their high boiling points. The residual solvents hinder the effective formation of vdW interfaces between the nanosheets, leading to increased contact resistance, which can degrade device performance.^[^ ^26^, ^27^, ^28^ ^]^ Furthermore, these solvents are not desirable for use in industrial applications because of their environmental toxicity and contamination issues. In this regard, it is imperative to identify alternative solvent systems that maintain the dispersion stability of the materials while supporting sustainable manufacturing procedures.^[^ ^29^, ^30^, ^31^ ^]^

Moreover, the use of multiple solvents during the conventional patterning process limits the optimization of device performance. Photolithography, a widely employed patterning method, inevitably introduces chemical residues, such as polymeric residues from photoresists, alkaline developers, and acidic etchants, which can significantly compromise the intrinsic properties and overall performance of 2D nanomaterial‐based electronics.^[^ ^32^ ^]^ Alternative approaches, such as the lift‐off process and adhesion energy‐engineered methods, frequently necessitate the implementation of a transfer process to successfully integrate vdW heterostructures or the preparation of orthogonal solvents that do not disrupt the underlying prepatterned layer.^[^ ^33^, ^34^ ^]^ Screen printing and inkjet printing also suffer from inherent limitations, such as low resolution, poor uniformity, and weak chemical robustness, which hinder their scalability and reliability.^[^ ^35^, ^36^, ^37^ ^]^ To overcome such limitations, a direct patterning approach that utilizes an ultraviolet (UV) light‐responsive cross‐linker has been proposed.^[^ ^4^ ^]^ However, this approach can only be implemented by preparing stable dispersions using chloroform, which is an undesirable solvent to use in practice because of its toxicity and environmentally harmful properties. Therefore, extensive research into strategies for patterning various 2D nanomaterials and integrating multiple layers using environmentally friendly green solvents is crucial for the successful practical implementation of advanced and versatile electronic devices based on solution‐processed 2D nanomaterials.

In this study, we developed a direct photopatterning method by preparing a stable dispersion of 2D nanosheets and UV light‐responsive cross‐linker molecules in isopropyl alcohol (IPA), which is a widely recognized eco‐friendly green solvent system. To optimize the alcohol‐based dispersion preparation, we thoroughly analyzed solvent systems using Hansen solubility parameters (HSPs) to identify a desirable solvent system for stabilizing both the 2D nanomaterials and the cross‐linker. Moreover, the cross‐linker ((oxybis(ethane‐2,1‐diyl))bis(oxy))bis(ethane‐2,1‐diyl) bis(4‐azido‐2,3,5,6‐tetrafluorobenzoate), called 2Bx‐4EO, enables the patterning and integration of 2D networks into scalable vertical vdW heterostructures in a photoresist‐free, orthogonal multilayer patterning manner without the need for polymers and acidic solvents. This method was applied to directly photopattern and assemble a series of 2D nanosheet dispersions in IPA, each with diverse electronic properties, into vertical vdW heterostructures such as field‐effect transistors (FETs), various logic gates (NOT, NAND, and NOR gates), and static random‐access memory (SRAM). For these demonstrations, a list of 2D nanosheets was used, including metallic graphene, *n*‐type semiconducting molybdenum disulfide (MoS_2_), *p*‐type semiconducting tungsten diselenide (WSe_2_), and hafnium disulfide (HfS_2_), which was thermally transformed into insulating hafnium dioxide (HfO_2_). The resulting FET arrays exhibited high spatial uniformity of electronic properties, with an average electron mobility (*µ*_e_) of 20.2 cm^2^ V^−1^ s^−1^, a threshold voltage of 2.0 V, and an on/off current ratio (*I*_ON_/*I*_OFF_) of 2.7 × 10^6^. We believe that this advanced strategy will pave the way for the development of high‐performance, environmentally friendly 2D nanomaterial‐based electronics at the wafer scale.

## 2 Results and Discussion

**Figure 1:**
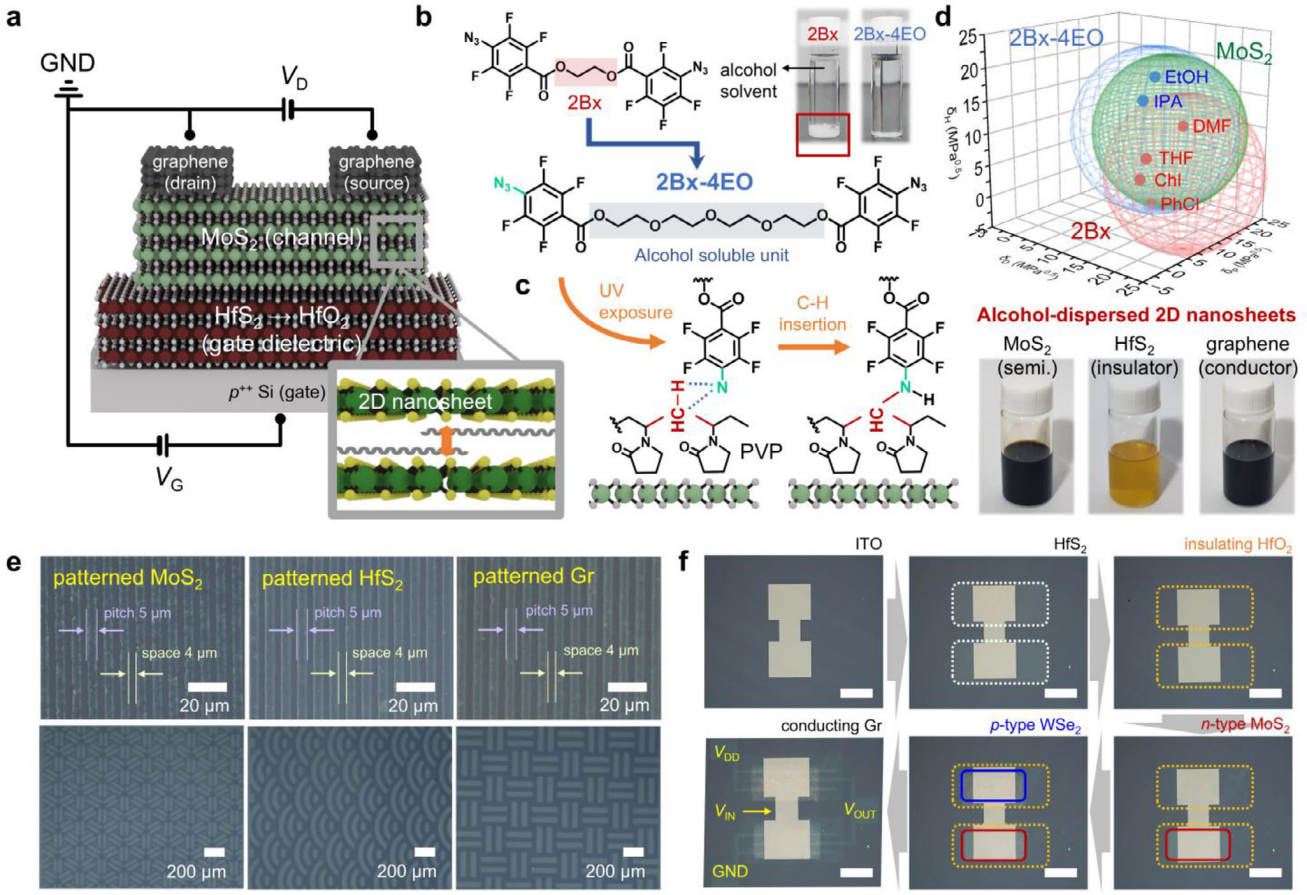
a) Schematic illustration of the FET based on fully photopatterned 2D nanomaterials—namely, graphene, MoS_2_, and HfO_2_ derived from HfS_2_ oxidation as the drain and source electrodes, semiconducting channel, and gate dielectric, respectively. The inset image depicts the atomic arrangement of the cross‐linked nanosheet interlayer within the MoS_2_ flakes. b) Chemical structures of 2Bx (red) and 2Bx‐4EO (blue), which were derived from 2Bx to enhance the solubility in green solvents. The photographic images show the solubility of 2Bx and 2Bx‐4EO in an alcohol‐based solvent. c) Photochemical reaction that ensures cross‐linking between neighboring PVP and the nitrene moiety of 2Bx‐4EO through C─H insertion. d) Hansen solubility space for 2Bx‐4EO (blue), 2Bx (red), and MoS_2_ (green). The blue‐filled circle indicates solvents common to both MoS_2_ and 2Bx‐4EO. The red‐filled circle represents solvents common to both MoS_2_ and 2Bx. The photographic images show the dispersed 2D nanomaterials in IPA. e) Optical microscopic images of the high‐resolution patterns (top) and various shapes (bottom) of the 2D nanomaterials. f) Fabrication process for the inverter gates and optical microscopic images of the component layers after each patterning step. Scale bar: 400 µm.

**Figure** **1**a presents a schematic illustration of the FET architecture based on fully photopatterned 2D nanomaterials, including metallic graphene, semiconducting MoS_2_, and insulating HfO_2_ derived from semiconducting HfS_2_ via thermal oxidation. The gray box highlights the atomic arrangement of a cross‐linked polyvinylpyrrolidone (PVP) within an MoS_2_ interlayer. To integrate these complex bottom‐gate top‐contact (BGTC) configurations, all components had to be precisely patterned while maintaining excellent chemical and mechanical robustness against exposure to various chemical substances throughout the fabrication process. As discussed earlier, the cross‐linking‐based patterning strategy allows for the precise patterning and compact integration of target materials without the need for orthogonal solvents or additional transfer processes. Moreover, this strategy simplifies device fabrication by directly stabilizing solution‐processed materials on the substrate, ensuring compatibility with a wide range of 2D nanomaterials and enhancing overall process efficiency. Therefore, the 2D nanomaterials dispersed in alcohol‐based solvents were cross‐linked using 2Bx‐4EO, a photocross‐linker containing photoreactive azide moieties bridged by four ethylene oxide (4EO) hydrophilic units, which ensure excellent miscibility with green solvents (Figure 1b). The 2Bx‐4EO cross‐linker was synthesized through an esterification reaction between 4‐azido‐2,3,5,6‐tetrafluorobenzoyl chloride and tetraethylene glycol. Detailed information regarding the synthetic procedure and thermal properties of 2Bx‐4EO is provided in the Methods section and supporting information (Figures S1–S5, Supporting Information). In contrast, another cross‐linker ethane‐1,2‐diyl bis(4‐azido‐2,3,5,6‐tetrafluorobenzoate), called 2Bx, which contains a short ethylene unit, does not dissolve in alcohol solvents (IPA), as demonstrated in the photographic images (red box in Figure 1b).

Upon exposure to 254 nm UV light, the fluorinated aryl azide groups in the cross‐linker undergo photolysis, generating highly reactive singlet nitrene and releasing inert nitrogen gas.^[^ ^28^, ^38^ ^]^ The as‐generated reactive nitrene intermediate readily undergoes C–H insertion reactions in the presence of nearby alkyl chains containing C─H bonds. As illustrated in Figure 1c, the phenyl azide terminal of the cross‐linker undergoes a C─H insertion reaction with the alkyl chains of PVP, a residual surfactant present in the 2D nanomaterial solutions. It is worth noting that PVP was added to stabilize the 2D nanosheets in green solvents, and despite the several centrifugation processes performed for purification, a small amount of PVP remained as a residual surfactant in the 2D nanomaterial solutions.^[^ ^4^, ^12^ ^]^ We exploited the alkyl chains of this residual PVP polymer as reaction sites for the abovementioned photochemical reaction.

To maximize the potential of this cross‐linking‐based patterning strategy for a wide range of 2D nanomaterials, a comprehensive evaluation of their miscibility in diverse chemical solvents was essential during the solution process. Selecting the optimal solvent required a thorough understanding of the cross‐linker–solvent and 2D nanosheet–solvent interactions, respectively. Accordingly, we performed an HSP analysis on the various 2D nanomaterials, 2Bx, and 2Bx‐4EO with respect to various chemical solvent candidates, as shown in Figure S6 (Supporting Information). The “like dissolves like” principle of the HSP analysis is commonly used to scale solvent–molecule interactions based on three parameters: the dispersive force (*δ*_D_), polar force (*δ*_P_), and hydrogen‐bonding force (*δ*_H_) between the molecules.^[^ ^39^, ^40^, ^41^ ^]^ The Hansen solubility spheres of 2Bx‐4EO, 2Bx, and MoS_2_ are represented in blue, red, and green, respectively, in Figure 1d. The corresponding Venn diagram (Figure S7, Supporting Information) categorizes the good and bad solvents for 2Bx‐4EO (blue), 2Bx (red), and MoS_2_ (green) based on the information acquired from the HSP analysis of 22 solvent candidates. The prevalent solvents that effectively dissolve both 2Bx and MoS_2_ are toxic and hazardous organic solvents, such as DMF, tetrahydrofuran (THF), chloroform (CF), and chlorobenzene (PhCl) (red circle in Figure 1d). However, the incorporation of 2Bx‐4EO significantly broadens the range of compatible solvents. Notably, 2Bx‐4EO and MoS_2_ are both compatible with environmentally friendly alcohol‐based green solvents, such as ethyl alcohol (EtOH) and IPA (blue circle in Figure 1d), demonstrating improved compatibility with sustainable solution process systems. Therefore, we employed 2Bx‐4EO as a cross‐linking agent instead of 2Bx to promote the development of eco‐friendly materials and processing technologies. Based on this optimized solubility analysis, we successfully synthesized a stable dispersion of 2D nanomaterials in IPA, as shown in the photographic images in Figure 1d. The detailed synthesis process is described in the Methods section. The atomic force microscopy (AFM) images and thickness of individual nanoflakes from the various solution‐processed 2D nanomaterials are shown in Figure S8 (Supporting Information), revealing that the 2D nanomaterials were sufficiently exfoliated and uniformly dispersed as single flakes in IPA solvent.

Figure 1e presents the optical microscopic images of the photopatterned 2D nanosheets arranged into high‐resolution patterns and various shapes through the photoinduced cross‐linking process, demonstrating the versatility and precision of this fabrication process. Notably, well‐defined multiple line patterns with a width of 5 µm and a gap of 4 µm were successfully achieved using this cross‐linking‐based patterning process. This result demonstrates that the photopatterned 2D networks maintain high uniformity due to the natural alignment of basal planes parallel to the underlying layer, as well as the sophisticated control achieved through the photopatterning process. Other various shapes of the photopatterned 2D nanosheets are shown in Figure S9 (Supporting Information). The fabrication procedures for complementary metal‐oxide semiconductor (CMOS) inverter gates composed of two different types of 2D semiconductors are depicted in Figure 1f to illustrate facile techniques for integrating vertically stacked vdW heterostructures. To construct a multilayer inverter architecture consisting of insulating HfO_2_ (oxidized from HfS_2_)/*n*‐type semiconducting MoS_2_ or *p*‐type semiconducting WSe_2_/conducting graphene heterostructures, we employed cross‐linking‐based patterning and multi‐stacking strategies. These strategies enable device fabrication on a single wafer without the need for orthogonal solvents or additional complicated transfer processes. Overall, these results indicate that this cross‐linking process holds tremendous promise for high‐resolution patterning and seamless integration into high‐throughput, solution‐based manufacturing processes.

**Figure 2:**
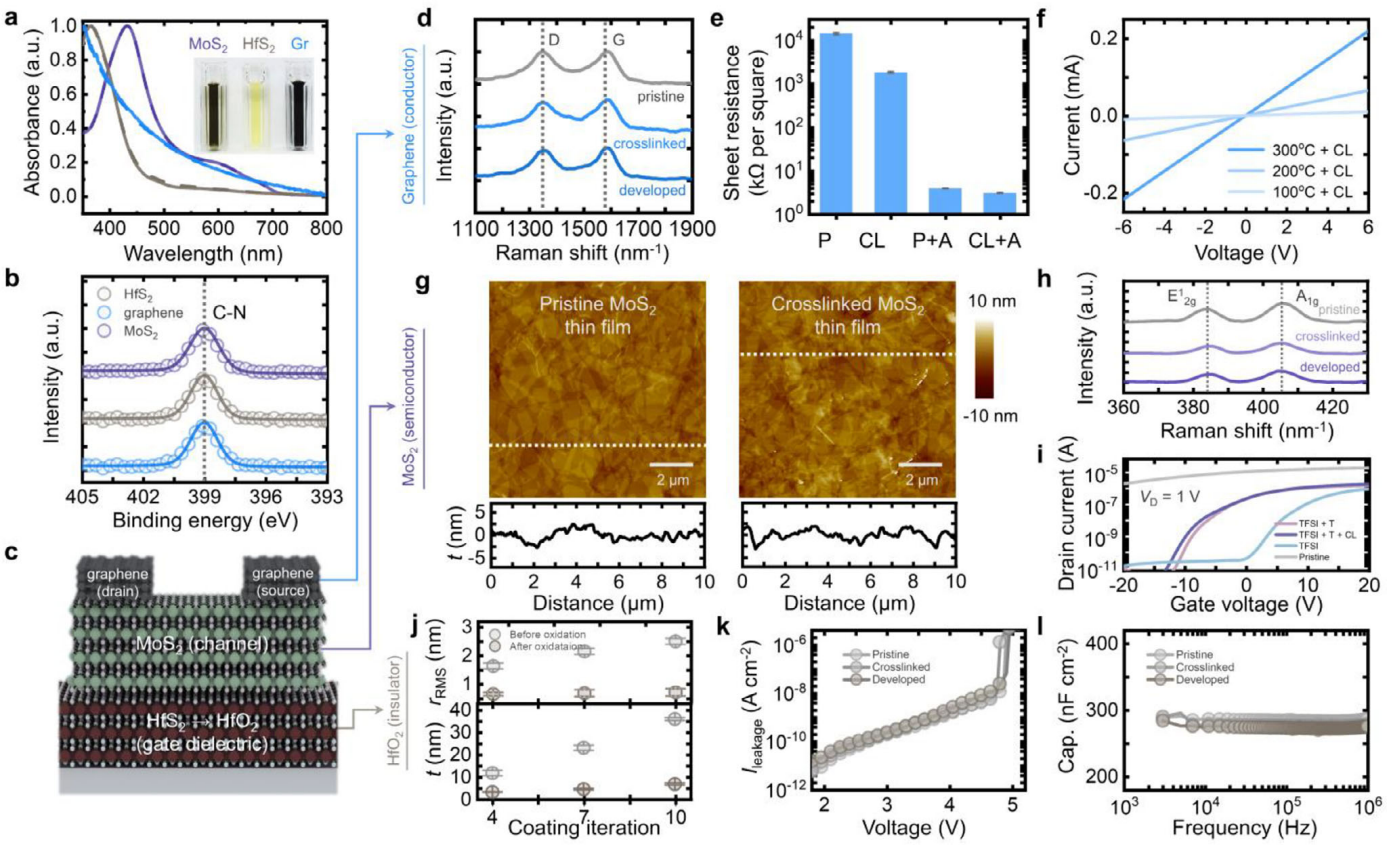
a) Absorbance spectra of the MoS_2_, HfS_2_, and graphene dispersions before (dashed lines) and after (solid lines) cross‐linker incorporation. The inset shows the photographic images of each dispersion. b) XPS spectra of MoS_2_, HfS_2,_ and graphene, showing a C–N peak indicative of PVP molecule adsorption onto nanosheets. c) Schematic illustration of the heterostructure device composed of the electronically distinct 2D thin films, fabricated using photocross‐linking. d) Raman spectra of the pristine, cross‐linked, and developed graphene thin films. e) Sheet resistance of graphene thin films based on photocross‐linking and thermal annealing. f) current‐voltage characteristics of the cross‐linked graphene at different annealing temperatures. g) AFM images and height profiles of the pristine and cross‐linked MoS_2_ thin films. h) Raman spectra of the pristine, cross‐linked, and developed MoS_2_ thin films. i) Transfer characteristics of MoS_2_ under different conditions, including TFSI treatment, thermal annealing, and photocross‐linking. j) Structural profiles of HfS_2_ thin films (before oxidation) and HfO_2_ thin films (after oxidation), showing the RMS roughness and height as functions of coating iterations. k) Breakdown voltage and l) frequency‐dependent areal capacitance of pristine, cross‐linked, and developed HfO_2_.

Based on the HSP analysis results, we employed 2Bx‐4EO as a photocross‐linker to achieve stable dispersions of the various 2D nanomaterials—including MoS_2_, HfS_2_, and graphene—even in alcohol‐based solvents. To experimentally validate this, we analyzed the UV–vis spectra of the 2D nanomaterial dispersions in IPA with and without the photocross‐linker to directly evaluate dispersion stability. As illustrated in **Figure** **2**a, the absorbance spectra exhibited negligible deviations before (dashed lines) and after (solid lines) cross‐linker incorporation, confirming that the dispersions remained stable even after the addition of the cross‐linker, consistent with the HSP analysis results presented in Figure 1d. Next, we investigated the presence of PVP on the surface of the 2D nanosheets, as it is a crucial component for inducing cross‐linking reactions with 2Bx‐4EO. Specifically, we conducted X‐ray photoelectron spectroscopy (XPS) measurements on the different 2D nanomaterials (Figure 2b), all of which exhibited a clear C─N binding peak. These results indicate that residual PVP remains on the surface of the 2D nanomaterials even after the rinsing process, thereby facilitating the C─H insertion reaction with 2Bx‐4EO—the key mechanism for cross‐linking. Based on these findings, we fabricated heterostructure FETs in a BGTC configuration using electronically distinct 2D thin films as the electrode, channel, and gate dielectric through photocross‐linking (Figure 2c). Prior to device implementation, we systematically characterized cross‐linked graphene (Figure 2d–f), MoS_2_ (Figure 2g–i), and HfO_2_ (Figure 2j–l) to assess their suitability as an electrode, a semiconducting channel, and a gate dielectric, respectively, and to optimize their material properties.

First, to examine the structural and electrical impacts of cross‐linking on graphene thin films (Figure S10, Supporting Information), we coated the dispersion onto a 300 nm‐thick SiO_2_/Si substrate. Figure 2d presents the Raman spectra of the differently processed graphene films—pristine, cross‐linked, and developed—each exhibiting distinct D and G bands, which confirm the preservation of sp^2^ carbon bonding.^[^ ^42^ ^]^ To quantify defect density, we further analyzed the D peak intensity to the G peak intensity (*I*_D_/*I*_G_) ratio, which decreased from ≈1.0 in the pristine film to ≈0.9 in the cross‐linked and developed films. This suggests that the cross‐linking process reduces defect density in the basal plane of graphene, likely due to photonic annealing effects induced by UV light exposure during the reaction.^[^ ^43^ ^]^ To further evaluate the electrical properties, we measured the sheet resistance of the differently processed films (Figure 2e). The measured average sheet resistance values were ≈1.4 × 10^4^ kΩ sq^−1^ (P: pristine), ≈1.8 × 10^3^ kΩ sq^−1^ (CL: cross‐linked), 3.9 kΩ sq^−1^ (P + A: pristine + annealed), and 3.0 kΩ sq^−1^ (CL + A: cross‐linked + annealed). These results indicate that the sheet resistance slightly decreased after cross‐linking and substantially decreased after thermal annealing. The initial improvement is primarily due to the removal of oxygen functional groups and enhancement in crystallinity induced by photonic annealing, as previously illustrated.^[^ ^4^, ^12^, ^37^ ^]^ The more pronounced improvement in sheet resistance is due to the enhanced nanosheet contact promoted through residual solvent removal by thermal annealing. To further investigate the evolution of electrical conductivity in the cross‐linked graphene films, we analyzed their current‐voltage characteristics at different annealing temperatures (Figure 2f). The drain current increased monotonically as the annealing temperature increased. Notably, the extracted electrical conductivity for graphene annealed at 300 °C was ≈3.6 × 10^3^ S m^−1^, which is comparable to that of previously reported solution‐processed graphene thin films after postprocessing.^[^ ^44^ ^]^

Similarly, we investigated the applicability of cross‐linked MoS_2_ as a semiconducting channel by cross‐linking a comprehensive analysis of its material properties. Figure 2g presents the AFM images of pristine and cross‐linked MoS_2_ thin films, revealing negligible morphological deviation. The root‐mean‐square (RMS) roughness extracted from the AFM line scans remained consistent at ≈1.3 nm, confirming that cross‐linking induces marginal structural changes (Figure S11, Supporting Information). Next, Raman spectroscopy was conducted to assess the influence of the cross‐linking reaction on the defect density and electronic states (Figure 2h). Regardless of their processing conditions, all MoS_2_ films exhibited characteristic E^1^_2g_ and A_1g_ peaks at 383.7 and 405.4 cm⁻¹, respectively. The absence of significant peak shifts indicates that the cross‐linking and subsequent development processes do not induce additional sulfur vacancies or alter the doping state of the material, preserving its intrinsic electronic properties. To directly evaluate electrical performance, we fabricated MoS_2_ FETs on a 50 nm‐thick Al_2_O_3_/Si^++^ substrate and measured their transfer characteristics (Figure 2i). The pristine MoS_2_ FET exhibited degenerated *n*‐doped behavior (gray curve), attributed to the significant concentration of sulfur vacancies created during solution processing.^[^ ^11^, ^45^ ^]^ After treatment with bis(trifluoromethane)sulfonimide (TFSI), an established sulfur vacancy healing method, the device regained typical *n*‐type semiconducting properties (sky blue curve).^[^ ^46^ ^]^ Additionally, to further optimize the electrical performance of the device, thermal treatment was conducted to reduce inter‐nanosheet resistance, which resulted in an increased drain current (pink curve). Notably, devices fabricated using the cross‐linked MoS_2_ thin film exhibited negligible deviation in performance (purple curve), demonstrating that cross‐linking does not exert a detrimental effect on electrical properties.

To extend the cross‐linking approach to the realization of a gate dielectric layer, we explored HfO_2_ formation through the thermal oxidation of HfS_2_. Thermal annealing caused the HfS_2_ nanosheets to convert into HfO_2_ while preserving their original 2D structure, resulting in a significant thickness reduction of ≈70–80%. To clarify the effect of thermal oxidation on the thin films, we investigated the structural characteristics of the cross‐linked HfS_2_ and oxidized HfO_2_ thin films using AFM (Figure 2j). The HfO_2_ films exhibited thickness reductions of 70–80% (Figure S12, Supporting Information), similar to that of single flakes, and maintained an RMS roughness of ≈0.7 nm, which is much smaller than the pre‐oxidation roughness (≈2.5) (Figures S13 and S14, Supporting Information), confirming that cross‐linking does not induce significant structural changes. We further evaluated dielectric properties by fabricating Au/HfO_2_/Si^++^ metal–insulator–metal capacitors. The coating iteration of HfO_2_ was set to 10 to achieve optimal performance. Figure 2k shows that the dielectric films displayed a breakdown voltage of 4.8 V, with no significant variation in performance across different processing conditions. Additionally, frequency‐dependent capacitance measurements revealed stable capacitance values of 286, 278, and 275 nF cm^−2^ for the pristine, cross‐linked, and developed HfO_2_ films, respectively, across the measured frequency range (Figure 2l).

**Figure 3:**
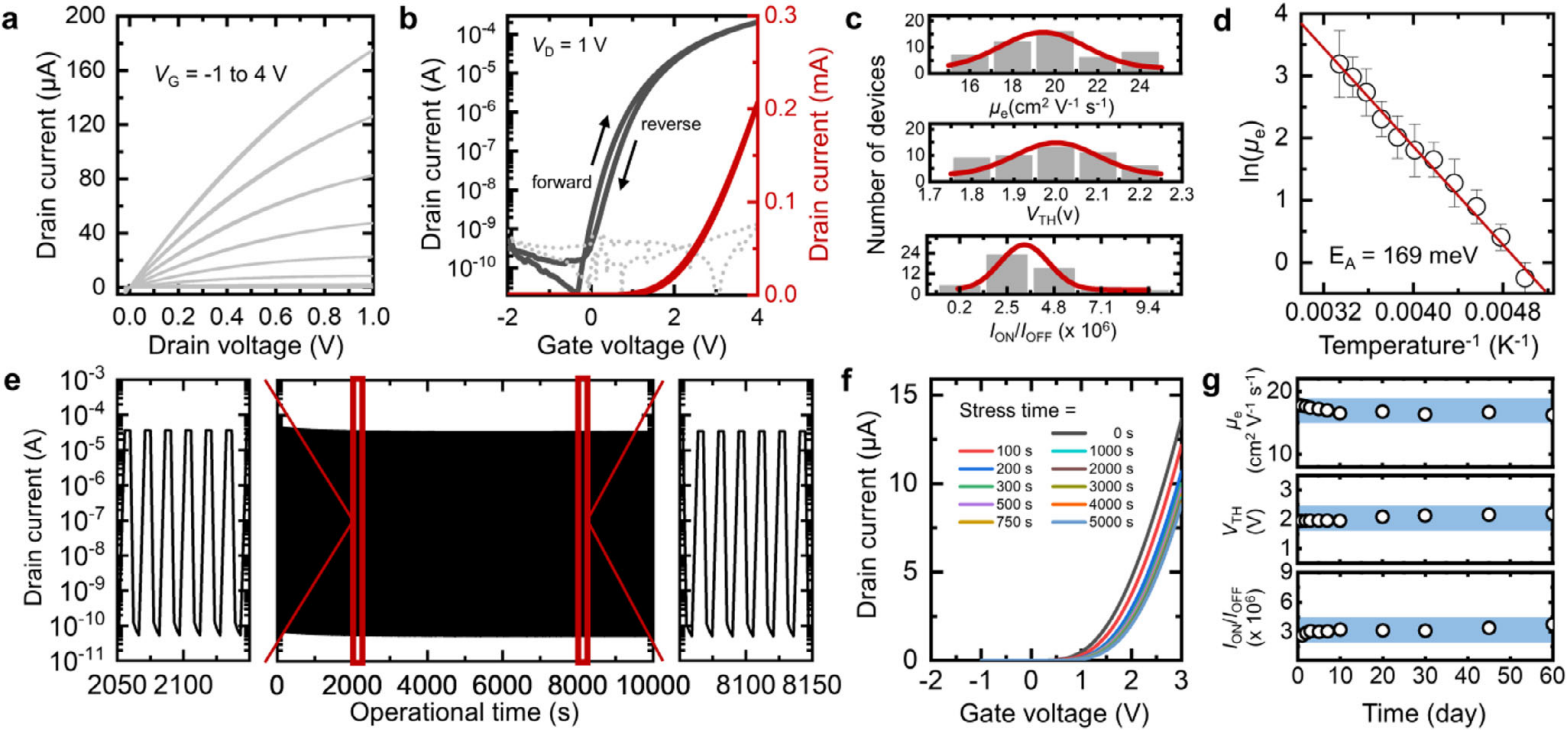
a) Representative output and b) Transfer characteristics of the fully photopatterned 2D nanomaterial‐based FETs cross‐linked using 2Bx‐4EO. The channel length and width of the MoS_2_‐based FETs are 50 and 1400 µm, respectively. c) Summary of the electron mobility (top), threshold voltage (middle), and on/off current ratio (bottom) of 49 FET arrays. d) Arrhenius plot of the temperature–mobility relationship for the cross‐linked MoS_2_ thin films. e) Operational stability of the 2D nanomaterial‐based FETs over 10000 s of periodic turn‐on and turn‐off cycles. f) Transfer curves of an FET prepared with cross‐linked 2D nanomaterials under prolonged gate bias stress in ambient conditions. g) Environmental stability of the FETs as a function of exposure time and deviations in electrical parameters.

Based on the comprehensive analysis of each cross‐linked 2D network, we designed and investigated the electrical characteristics of FETs incorporating fully photopatterned 2D nanomaterials—graphene, MoS_2_, and HfO_2_ (oxidized from HfS_2_)—with the BGTC configuration (All components were cross‐linked using only 5 wt.% of 2Bx‐4EO. More detailed fabrication procedure is discussed in the Methods section). **Figure** **3**a shows the drain current (*I*_D_) versus drain voltage (*V*_D_) of the 2D nanomaterial‐based FETs at various gate voltages (*V*_G_) ranging from −1 to 4 V. These output characteristics exhibit clear gate modulation, displaying both linear and saturation behavior with respect to the *V*_D_ region. In particular, the linear behavior observed at low *V*_D_ regions clearly indicates the formation of a conformal contact between the MoS_2_ channel and the graphene electrodes. Figure 3b presents the representative electrical transfer characteristics (i.e., *I*_D_ versus *V*_G_) on both a logarithmic scale (black) and a linear scale (red), along with the gate leakage current (*I*_G_, dashed gray line) at a fixed *V*_D_ of 1 V. The increase in *I*_D_ with a positive shift in *V*_G_ clearly demonstrates the typical behavior of an *n*‐type transistor. Moreover, the 2D nanomaterial‐based FET exhibited superior electrical characteristics, achieving the highest *µ*_e_ value of 23.89 cm^2^ V^−1^ s^−1^, which was calculated using the equation *I*_D_  =  (*W* ^.^ *L* ^−1^) (*V*_D_^.^ *µ*_e_^.^ *C*_i_) (*V*_G_ – *V*_TH_), where *W* is the channel width (1400 µm), *L* is the channel length (50 µm), *C*_i_ is the effective capacitance of the gate dielectric (275 nF cm^−2^ from Figure 2l), and *V*_TH_ is the threshold voltage.^[^ ^11^ ^]^ The thickness of the MoS_2_ thin film is confirmed as almost 10 nm, as shown in Figure S15 (Supporting Information).

Furthermore, the high‐throughput capability of the cross‐linking‐based patterning process and the excellent spatial uniformity, which is one of the key advantages of 2D nanosheets synthesized through molecular intercalation‐based electrochemical exfoliation methods, were demonstrated by the successful fabrication of an array with 49 FETs (Figure S16, Supporting Information). A corresponding photographic image of the 49‐FET array is provided in Figure S17 (Supporting Information). The representative electrical parameters, including *µ*_e_, *V*_TH_, and *I*_ON_/*I*_OFF_, were calculated from the transfer curves of all 49 FETs based on the 2D nanomaterials. As depicted in Figure 3c, the array exhibited a high spatial uniformity of their electrical characteristics, with an average *µ*_e_ of 20.2 cm^2^ V^−1^ s^−1^, a *V*_TH_ of 2.0 V, and an *I*_ON_/*I*_OFF_ ratio of 2.7 × 10^6^, all showing minimal deviation. In addition, Table S1 (Supporting Information) provides a comparison between our photopatterned MoS_2_ FETs and previously reported devices.

To elucidate the origin of the high *µ*_e_ of over 20 cm^2^ V^−1^ s^−1^, the temperature‐dependent transfer characteristics of the photopatterned 2D nanomaterial‐based FETs were measured. Figure S18 (Supporting Information) depicts a series of transfer curves for a photocross‐linked 2D nanomaterial‐based single FET device at different temperatures (*T*) ranging from 300 to 200 K, with a 10 K reduction at each step. The extracted *µ*_e_ values at each *T* exhibited a decreasing trend as the *T* approached 200 K, implying that electron transport in MoS_2_ nanosheets follows an Arrhenius relationship, *µ* = *µ*_0_^.^exp(−*E*_a_/*k*_B_ *T*), where *µ* is the carrier mobility, *µ*_0_ is the pre‐exponential mobility, *E*_a_ is the activation energy for charge transport, and *k*_B_ is the Boltzmann constant (8.617 × 10^−5^ eV K^−1^).^[^ ^11^, ^47^, ^48^ ^]^ Figure 3d shows the Arrhenius plot illustrating the relationship between *µ*_e_ and *T* ^−1^; an *E*_a_ value of 169 meV was extracted from the slope of this plot. Noteworthy, the positive correlation between mobility and temperature indicates thermally assisted hopping‐type transport in the thin film. This transport mechanism is common in solution‐processed MoS_2_ nanosheet systems because the charge carriers in the nanosheets require additional energy to overcome interconnect barriers within the channel.^[^ ^49^ ^]^ Nevertheless, the extracted *E*_a_ values of our cross‐linking‐based patterned MoS_2_ FET are considerably lower than other reported values, indicating minimal energy barriers, which result in superior electrical properties. We attribute the low *E*_a_ values to the following factors: 1) Molecular intercalation‐based electrochemically exfoliated MoS_2_ nanosheets exhibit well‐ordered alignment, with their basal planes oriented parallel to the underlying layer. This orientation enables the formation of plane‐to‐plane vdW contacts between adjacent layers. 2) The 2Bx‐4EO cross‐linker facilitates the formation of a photoinduced cross‐linking network at the nanosheet‐to‐nanosheet interfaces, enhancing both charge transport and the integration density of the vertically stacked vdW thin‐film networks. Overall, we have successfully fabricated high‐performance, high‐uniformity FETs based on 2D nanomaterials using the cross‐linker.

The stability of the fully photopatterned 2D nanomaterial‐based FET was comprehensively evaluated by monitoring the transfer curves under diverse extreme conditions. First, the operational stability of the electronics was tested under repeated on and off states, as shown in Figure 3e. Input voltages were applied to the device by alternating *V*_G_ between −1 V (off state) and 3 V (on state) at a constant *V*_D_ of 1 V for ≈10,000 s. During the operational stability test, no significant degradation was observed, as there were only slight deviations in the on‐current and off‐current of the device. To further investigate the electrical stability of the device, a bias‐stress test was performed by monitoring the shift in *V*_TH_ under a constant *V*_G_ of 3 V over time, as shown in Figure 3f. The cross‐linked device demonstrated only a small magnitude *V*_TH_ shift even after being subjected to bias stress for 5,000 s (Figure S19, Supporting Information). Finally, an environmental stability test was conducted under ambient conditions for 60 days (Figure S20, Supporting Information), and the extracted electrical parameters are summarized in Figure 3g. The *µ*_e_, *V*_TH_, and *I*_ON_/*I*_OFF_ exhibited negligible changes even after 60 days of exposure to air, confirming the excellent air stability of the cross‐linked 2D networks.

**Figure 4:**
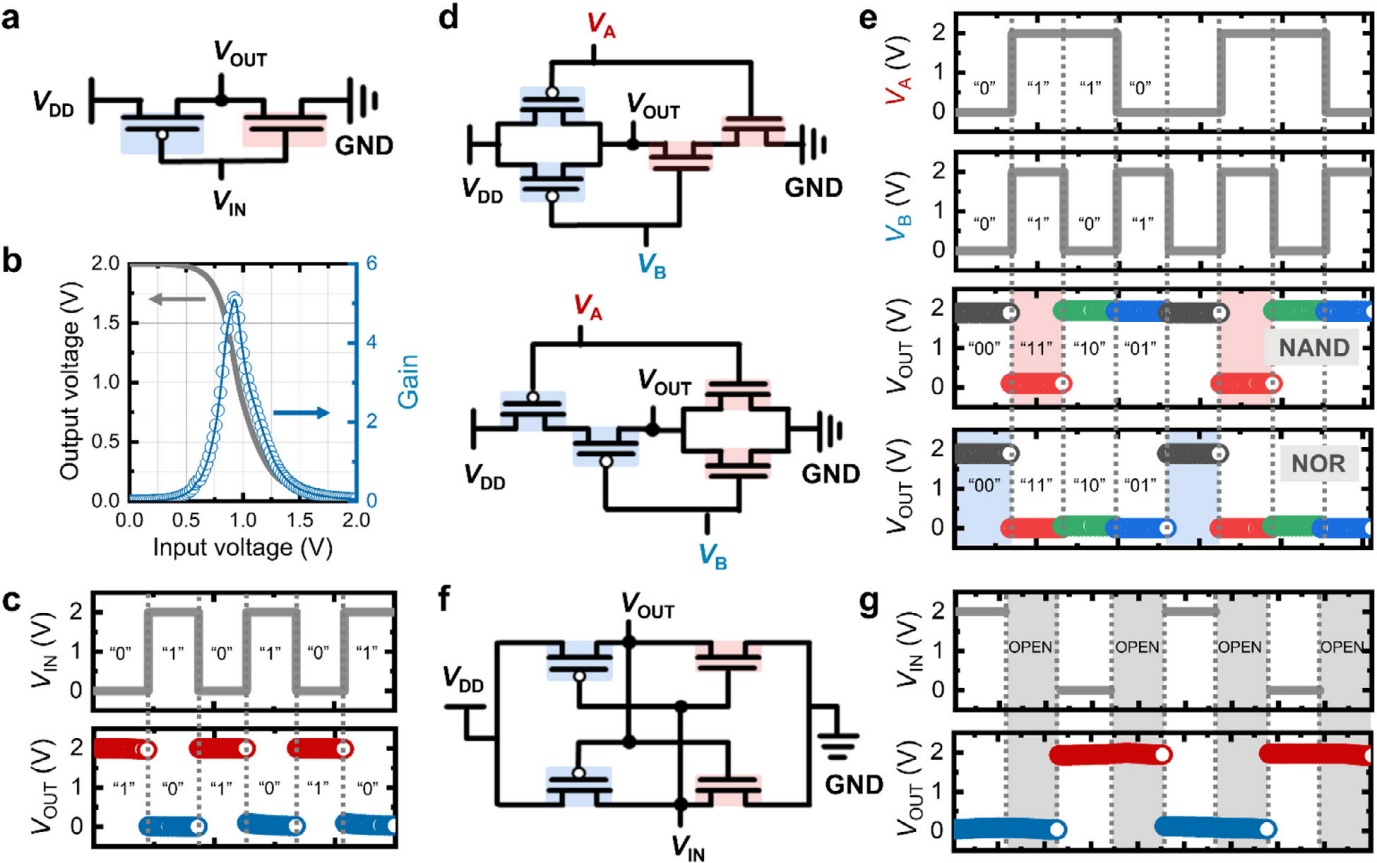
a) Electrical circuit diagram for the NOT logic gate. b) Corresponding voltage transfer curves and signal inverter gain. c) Input voltages and output results of the NOT logic gate under repeated operation. d) Electrical circuit diagram for the NAND logic gate (top) and NOR logic gate (bottom). e) Four possible input combinations and corresponding output voltages for the NAND and NOR gates. f) Schematic illustration of the SRAM. g) Operation of the SRAM based on the input condition. The gray regions indicate electrically open states.

Based on this, we integrated two types of electronic devices—an *n*‐type MoS_2_ FET and a *p*‐type WSe_2_ FET—to implement multiple CMOS logic circuits, including NOT, NAND, and NOR gates. We emphasize that the distinguished advantage of employing a photocross‐linking‐based patterning process to fabricate electronic devices is its simplicity, which allows for the seamless integration of various solution‐processed 2D nanomaterials without requiring orthogonal solvents or additional transfer processes. Consequently, the NOT gate structure (inverter) was easily fabricated by connecting the *p*‐type WSe_2_ FET and *n*‐type MoS_2_ FET in series on a single substrate, as shown in **Figure** **4**a and Figure S21 (Supporting Information). The transfer curve of the WSe_2_‐based FET is shown in Figure S22 (Supporting Information). The voltage transfer characteristics of the CMOS inverter were analyzed by measuring the output voltage (*V*_OUT_) as a function of the input voltage (*V*_IN_) at a fixed drain voltage (*V*_DD_) of 2 V (Figure 4b). The voltage transfer curve demonstrates that when *V*_IN_ is at a low level (0 V), *V*_OUT_ remains at a high level (2 V). As *V*_IN_ increases to a high level (2 V), *V*_OUT_ decreases to a low level (0 V), confirming that the NOT logic gate successfully exhibits the logical inversion of the signal input. The signal gain value of the inverter, defined as |*∂V*_OUT_/*∂V*_IN_|, was calculated to be over 5.2 from the corresponding voltage transfer curve (blue line in Figure 4b). When a *V*_IN_ with a logic state of “0” (0 V) is applied, the *V*_OUT_ yields the logic state “1” (2 V), and vice versa, indicating that the inverter demonstrates an ideal logic functionality even under repeated operation, as shown in Figure 4c.

Additional logic gates with more complicated circuit structures, namely, NAND and NOR, were also successfully designed and fabricated by assembling four independent FETs. The CMOS NAND gate was fabricated by connecting two *p*‐type WSe_2_ FETs in parallel and two *n*‐type MoS_2_ FETs in series, whereas the CMOS NOR gate was fabricated by connecting two *p*‐type WSe_2_ FETs in series and two *n*‐type MoS_2_ FETs in parallel (Figure 4d, top and bottom panels, respectively). The optical microscopic images of the NAND and NOR gates are presented in Figure S23 (Supporting Information). Both the NAND and NOR logic gates receive a combination of two *V*_IN_s (*V*_A_ and *V*_B_) and produce two distinct *V*_OUT_ values with either a high level (2 V) or a low level (0 V). The four possible input combinations of “*V*_A_,*V*_B_” were “0,0,” “1,1,” “1,0,” and “0,1,” as illustrated in Figure 4e. In this setup, both *V*_A_ and *V*_B_ were alternated between 0 V (logic state “0”) and 2 V (logic state “1”) while the *V*_DD_ was kept constant at 2 V. The corresponding *V*_OUT_ results of the NAND and NOR gates for each combination of *V*_A_ and *V*_B_ are also presented in Figure 4e. For the NAND gate, *V*_OUT_ remained at a low level (0 V) only for the “1,1” input combination (red region). In contrast, for the NOR gate, *V*_OUT_ reached a high level (2 V) only for the “0,0” logical input combination (blue region). Both complex logic circuits, i.e., the NAND and NOR gates, successfully executed ideal logic functions for all possible input combinations even under repeated operation.

Furthermore, two NOT gates were integrated to construct a more advanced architecture, namely, an SRAM, where the output of each inverter is connected to the input of the other (see Figure 4f). When a *V*_IN_ is applied to one inverter, its produced *V*_OUT_ is transmitted to the other, creating a feedback loop that causes the mutually connected inverters to continuously interact and sustain their opposing output states. Figure 4g shows the SRAM operation under different time variations, demonstrating that the *V*_OUT_ state remains stable even after *V*_IN_ is completely disconnected (open state, gray regions), even with repeated operation. These results highlight the applicability of the cross‐linking‐based patterning strategy, which can be extended to the integration of various 2D nanomaterials.

## 3 Conclusion

In this work, we introduce a photoresist‐free, cross‐linking‐based photopatterning strategy that enables sustainable green solvent‐based processes for the scalable fabrication of electronic devices using solution‐processed 2D nanomaterials. By optimizing the dispersion stability of both the 2D materials and photoresponsive cross‐linkers in eco‐friendly alcohol through HSP analysis, we achieved high‐resolution patterns with high chemical and mechanical stability, which ensured the reliable integration of vertically stacked vdW heterostructures. The fabricated FET arrays exhibited high spatial uniformity with electrical properties, displaying an average carrier mobility of >20 cm^2^ V^−1^ s^−1^ and excellent stability against repeated bias stress or air exposure under ambient conditions. Additionally, various logic gates and an SRAM were successfully implemented, demonstrating the versatility and scalability of this photopatterning strategy. These findings pave the way for the development of next‐generation electronic devices using environmentally sustainable 2D nanomaterial processing techniques, offering a promising alternative route to conventional photolithography patterning.

## 4 Experimental Section

### Synthesis of 2Bx‐4EO

Anhydrous dichloromethane and anhydrous pyridine were purchased from Sigma‐Aldrich. Tetraethylene glycol and 4‐azido‐2,3,5,6‐tetrafluorobenzoic acid were purchased from TCI. Thionyl chloride, anhydrous MgSO_4_, and all other solvents were purchased from Daejung. First, 4‐azido‐2,3,5,6‐tetrafluorobenzoic acid (2.2 g, 9.21 mmol) was combined with thionyl chloride (0.7 mL, 9.21 mmol) in anhydrous dichloromethane (10.0 mL) and heated to 70 °C for 18 h. After cooling, the solvent was removed, and the resulting 4‐azido‐2,3,5,6‐tetrafluorobenzoyl chloride was dissolved in anhydrous dichloromethane (10.0 mL). This solution was then added to a mixture of tetraethylene glycol (0.6 g, 3.07 mmol) and anhydrous pyridine (0.3 mL, 9.21 mmol) in anhydrous dichloromethane (15 mL). The reaction proceeded at room temperature for 8 h before being quenched with water (15 mL). The mixture was extracted using dichloromethane (15 mL) three times, and the combined organic layers were washed with brine (10 mL), dried over anhydrous MgSO_4_, and filtered. After solvent removal, the crude product was purified through silica gel column chromatography using ethyl acetate/n‐hexane (1:2) as the eluent. The final product, 2Bx‐4EO, was obtained as a yellow liquid (1.7 g, 86%). ^1^H NMR (400 MHz, CDCl_3_): *δ* = 4.52–4.50 (t, 4H), 3.82–3.79 (t, 4H), 3.69–3.64 (m, 8H). ^13^C NMR (100 MHz, CDCl_3_): *δ* = 159.43–159.35 (m), 146.80–146.78 (m), 144.28–144.10 (m), 141.89–141.88 (m), 139.43–139.22 (m), 123.63–123.39 (m), 107.96–107.66 (m), 70.80 (s), 68.85 (s), 65.69 (s). ^19^F NMR (376 MHz, CDCl_3_): *δ* = −138.49 to −138.59 (m, 4F), −150.95 to −151.05 (m, 4F). ESI‐MS *m*/*z*: [M + H] calcd, 628.10; observed, 629.10.

### Bulk Crystal Synthesis

Bulk MoS_2_ crystals were purchased from HQ Graphene. HfS_2_ crystals were synthesized using the chemical vapor transport method.^[^ ^12^ ^]^ Graphite foils were purchased from Thermo Fisher Scientific.

### Solution Processing

Tetraheptylammonium bromide (THAB; [CH_3_(CH_2_)_6_]4N(Br), 99%), tetrabutylammonium bisulfate (TBAB; [(C₄H₉)₄N]HSO₄, 99%), TFSI, sodium hydroxide (NaOH), acetonitrile (anhydrous, 99.8%), PVP (40 000 g mol^−1^), and 1,2‐dichloroethane (anhydrous, 99.8%) were purchased from Sigma‐Aldrich. IPA (99.5%) and DMF (99.5%) were purchased from Daejung Chemicals.

To prepare the MoS_2_ and HfS_2_ nanosheet dispersions, bulk crystals of MoS_2_ and HfS_2_ were electrochemically exfoliated through molecular intercalation. The electrolyte was prepared by dissolving THAB in 70 mL of acetonitrile at a concentration of 5 mg mL^−1^. The MoS_2_ crystal was immersed in the electrolyte using an alligator clip, while the HfS_2_ crystal was wrapped in a copper mesh before being clipped with an alligator clip. The crystals were used as the cathode, and a graphite electrode was used as the anode. A 7 V DC was applied for 1.5 h to facilitate intercalation. After intercalation, the MoS_2_ crystal was mildly rinsed with ethanol before being collected. Intercalated HfS_2_ was collected by filtering the electrolyte and intercalated crystal sediments through a membrane filter (nylon‐polyamide) using vacuum filtration. The collected crystals were then sonicated in PVP solution (22 mg mL^−1^ in DMF) for 30 min. Subsequently, centrifugation was performed at 4000 rpm for 10 min to eliminate unexfoliated crystals. The supernatants were collected and rinsed with IPA three times. Finally, another centrifugation was performed at 4000 rpm for 10 min, and the supernatants were collected, resulting in a high‐quality dispersion. To prepare the graphene nanosheet dispersion, graphite foils were cut into small pieces (3 cm × 1 cm) and used as both the anode and cathode. The electrolyte was prepared by dissolving 0.005 mol of TBAB in 53 mL of deionized water. The pH was adjusted to 7 by adding NaOH. For intercalation, a 10 V DC was applied to the electrodes for 1 h. The electrolyte beaker was placed in an ice bath to avoid overheating. The intercalated graphite foils were collected via vacuum filtration and rinsed with deionized water, followed by ethanol. The collected foils were sonicated in DMF for 30 min and centrifuged at 3000 rpm for 10 min to eliminate unexfoliated graphite foils. The supernatant was collected and rinsed in IPA three times. PVP was added to achieve a graphene‐to‐PVP weight ratio of 2:1. Finally, the dispersion was centrifuged at 4000 rpm for 10 min to eliminate any remaining graphite particles.

### Fabrication of the BGTC FET and Logic Gate Arrays

The BGTC FETs were fabricated on a doped Si wafer containing a thermally grown 100 nm‐thick SiO_2_ layer and a prepatterned indium tin oxide (ITO) substrate (SiO_2_/ITO). The SiO_2_/ITO substrate was cleaned via ultrasonication with acetone, IPA, and deionized water in sequence for 30 min each. Blend solutions of HfS_2_ and 2Bx‐4EO (5 wt.%) in IPA (anhydrous, 99.5%, Sigma‐Aldrich) were spin‐coated 10 times onto the substrate at 2500 rpm for 60 s. The as‐deposited HfS_2_ thin film was exposed to UV light (254 nm, 1000 W cm^−2^) for 5 s through a photomask. After the photochemical reaction, the cross‐linked HfS_2_ thin films were sonicated in fresh IPA solvents to remove the unexposed regions, and then they were annealed at 500 °C for 6 h under ambient conditions to form oxidized HfO_2_. Subsequently, blend solutions of *n*‐type MoS_2_ and 2Bx‐4EO (5 wt.%) in IPA were spin‐coated twice onto the patterned HfO_2_ layer to form a semiconducting channel. The same cross‐linking‐based patterning process was employed to expose the MoS_2_ thin films to UV light through a photomask, and the unexposed MoS_2_ regions were removed through sonication in fresh IPA. After annealing cross‐linked MoS_2_ thin films at 80 °C for 2 min to remove the residual IPA solvent, blend solutions of *p*‐type semiconducting WSe_2_ and 2Bx‐4EO in IPA were spin‐coated four times onto the patterned HfO_2_ layer for CMOS logic gates. The resulting WSe_2_ thin film was irradiated with a UV lamp for 5 s through a photomask, ensuring exposure only in the HfO_2_ regions where MoS_2_ was not patterned. The same rinsing process was applied to the film. The patterned MoS_2_ and WSe_2_ films were functionalized by being dipped in a TFSI solution (95%, Sigma‐Aldrich) in 1,2‐dichloroethane (anhydrous, 99.7%, Sigma‐Aldrich) (10 mg mL^−1^) for 30 min. Thereafter, they were thermally annealed at 250 °C for 30 min in an argon‐filled glove box. To form the electrode, a conducting graphene solution containing 2Bx‐4EO in IPA was spin‐coated three times onto the HfO_2_/MoS_2_ and HfO_2_/WSe_2_ multilayers on a single substrate and subsequently patterned using the same cross‐linking‐based process. The resulting electronic devices, which comprised both *p*‐type WSe_2_ and *n*‐type MoS_2_ FETs, were then annealed on a hot plate at 300 °C for 30 min in an argon‐filled glove box.

### Characterization

The molecular structure of 2Bx‐4EO was characterized using ^1^H NMR, ^13^C NMR, and ^19^F NMR spectroscopy on a Bruker Avance III 400 MHz and an Agilent 400 MHz FT‐NMR spectrometer with CDCl_3_ as the solvent. The mass spectrum of 2Bx‐4EO was acquired using electrospray ionization‐mass spectrometry (ESI‐MS) on an Accu TOF 4G+ Dart instrument. Thermal analysis was conducted using differential scanning calorimetry (DSC) on a TA Instruments DSC Q2000 with Tzero aluminum hermetic pans. The heating and cooling rates were set at 10 °C min^−1^ for the DSC measurements. The surface morphology of the 2D networks was characterized using optical microscopy (ECLIPSE LV100ND POL/DS, Nikon) and AFM (NX10, Park Systems) under ambient conditions. The electrical conductivity of the graphene and cross‐linked graphene thin films was measured using the four‐point probe method (CMT‐SR2000N, AIT). The specific capacitance of HfO_2_ and cross‐linked HfO_2_ was measured using an inductance–capacitance–resistance (LCR) meter (Agilent E4980A, Keysight), and the breakdown voltage was measured using a semiconductor characterization system (4200A‐SCS, Keithley) integrated with a vacuum chamber probe station (M6VC, MS Tech) The electrical characteristics of the FETs, fundamental logic gates, and SRAM were investigated using a Keithley 4200A‐SCS unit at 25 °C under a vacuum condition (under 10^−4^ Torr).

## Conflict of Interest

The authors declare no conflict of interest.

## Author Contributions

I.C.K., S.‐J.K., and W.H.C. contributed equally to this work. J.H.C., J.K., and B.K. initiated and supervised the entire research. I.C.K. and S.‐J.K. conducted and designed most of the experimental work and data analysis. J.K., S.K., Y.A.K., and J.K. assisted with the data analysis. W.H.C., V.M., Z.S., and Y.H. synthesized the materials. M.S.K. assisted with the manuscript writing. All authors discussed the progress of the research and contributed to the editing of the manuscript.

## Supporting information



Supporting Information

## Data Availability

The data that support the findings of the research are available from the corresponding author upon reasonable request.
