# Supplementary material for: Direct Photopatterning of Green Solvent‐Processed 2D Nanomaterials for Wafer‐Scale Electronics
Source: Adv Mater. 2025 Jul 21;37(40):e05917. doi: 10.1002/adma.202505917 (PMC12510278; doi:10.1002/adma.202505917)
Supplement: Supplementary file 1 — Supporting Information [file ADMA-37-e05917-s001.docx]

Supporting Information

**Direct Photopatterning of Green Solvent-processed 2D Nanomaterials for Wafer-scale Electronics**

In Cheol Kwak^1,†^, Se-Jin Kim^2,†^, Wan Ho Cho^3,†^, Jihyun Kim^1^, Seonkwon Kim^1^, Yonghyun Albert Kwon^1^, Vlastimil Mazánek^4^, Zdeněk Sofer^4^, Jinho Keum^5^, Yuchan Heo^3^, Moon Sung Kang^5,6^, BongSoo Kim^3,7,8*^, Joohoon Kang^1,*^, and Jeong Ho Cho^1,*^

^1^Department of Chemical and Biomolecular Engineering, Yonsei University, Seoul 03722, Republic of Korea

^2^School of Advanced Materials Science and Engineering, Sungkyunkwan University (SKKU), Suwon 16419, Republic of Korea

^3^Department of Chemistry, Ulsan National Institute of Science and Technology (UNIST), Ulsan 44919, Republic of Korea

^4^Department of Inorganic Chemistry, University of Chemistry and Technology Prague, Technicka 5, 166 28 Prague 6, Czech Republic

^5^Department of Chemical and Biomolecular Engineering, Sogang University, Seoul 04107, Republic of Korea

^6^Institute of Emergent Materials, Ricci Institute of Basic Science, Sogang University, Seoul 04107, Republic of Korea

^7^Graduate School of Semiconductor Materials and Device Engineering, Ulsan National Institute of Science and Technology (UNIST), 50 UNIST-gil, Ulsan 44919, Republic of Korea

^8^Graduate School of Carbon Neutrality, Ulsan National Institute of Science and Technology (UNIST), 50 UNIST-gil, Ulsan 44919, Republic of Korea

*Correspondence should be addressed to [bongsoo@unist.ac.kr](mailto:bongsoo@unist.ac.kr) (B.K.), [joohoon@yonsei.ac.kr](mailto:joohoon@yonsei.ac.kr) (J.K.), and [jhcho94@yonsei.ac.kr](mailto:jhcho94@yonsei.ac.kr) (J.H.C.).

| Fabrication  method | Channel  material | Solvents | Patterning methods | Mobility  (cm^2^ V^-1^ s^-1^) | Ref |
| --- | --- | --- | --- | --- | --- |
| Solution-processed  exfoliation | MoS_2_ | DMF | Photolithography | 5 | [1] |
| Solution-processed  exfoliation | MoS_2_ | DMF | Inkjet printing | - | [2] |
| Solution-processed  exfoliation | MoS_2_ | NMP | Spray coating | 0.22 | [3] |
| Solution-processed  exfoliation | MoS_2_ | NMP | - | 1.2 | [4] |
| Solution-processed  exfoliation | MoS_2_ | NMP | - | 12.5 | [5] |
| Solution-processed  exfoliation | MoS_2_ | Mixture of  IPA, 2-butanol | Inkjet printing | 0.27 | [6] |
| Solution-processed  exfoliation | MoS_2_ | Mixture of  IPA, 2-butanol | Inkjet printing | 11 | [7] |
| Solution-processed  exfoliation | MoS_2_ | Mixture of  Terpineol / EtOH | Inkjet printing | - | [8] |
| Solution-processed  exfoliation | MoS_2_ | Mixture of  H2O / EtOH / IPA | Printing | 9.85 | [9] |
| Solution-processed  exfoliation | MoS_2_ | IPA | Photolithography | 1.8 | [10] |
| Solution-processed  exfoliation | MoS_2_ | IPA | Photolithography | 5 | [11] |
| Solution-processed  exfoliation | MoS_2_ | IPA | Langmuir-Schaefer  deposition | 10.7 | [12] |
| Solution-processed  exfoliation | MoS_2_ | IPA | Photolithography | 13 | [13] |
| **Solution-processed**  **exfoliation** | **MoS_2_** | **IPA** | **Photo patterning** | **20.2** | **This**  **work** |

Table S1. Summary of FET performance metrics from MoS_2_-based devices fabricated using solution-processed exfoliation methods with various solvents and patterning methods.


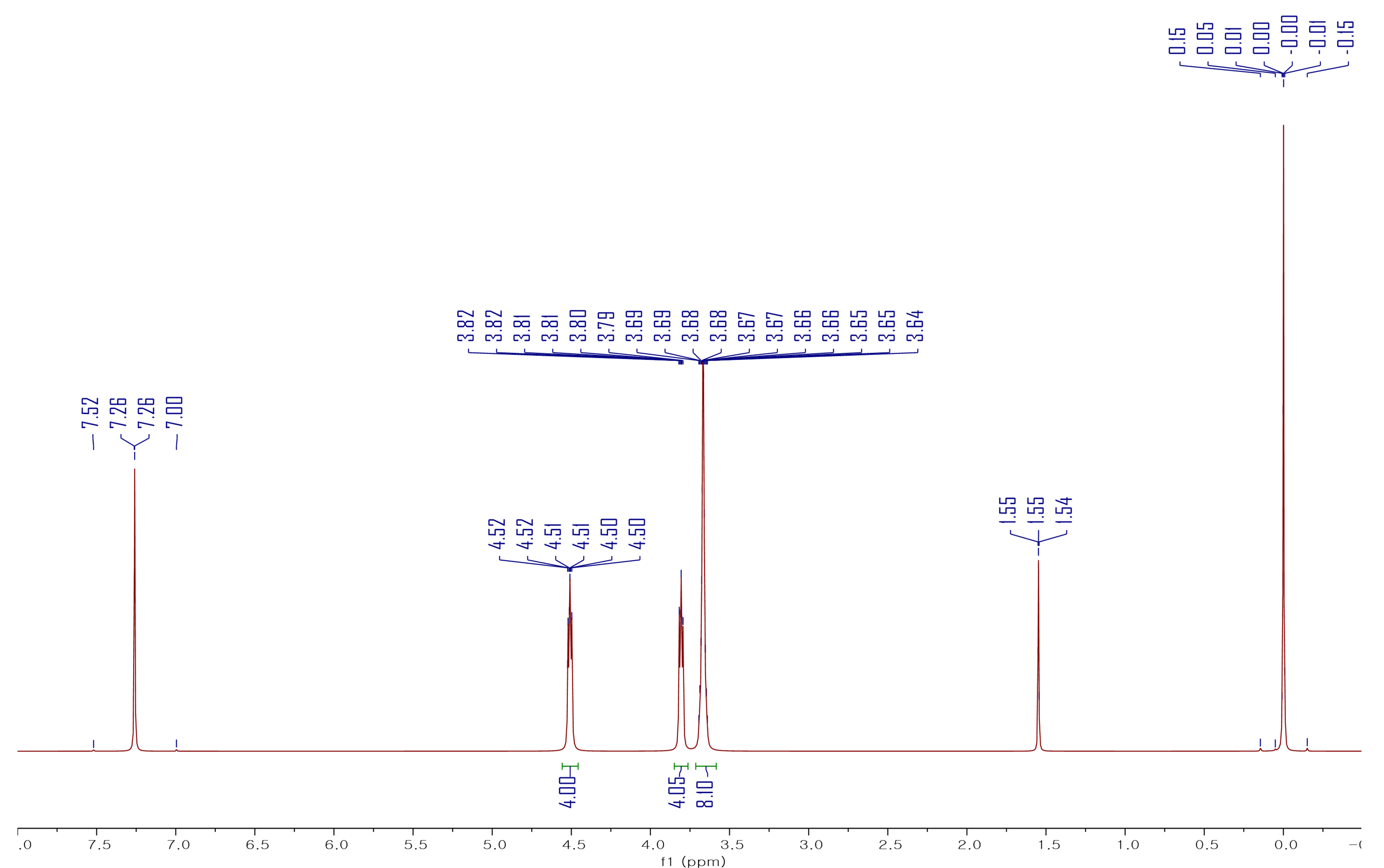


**Figure S1.** ^1^H NMR spectrum of 2Bx-4EO in CDCl_3_.


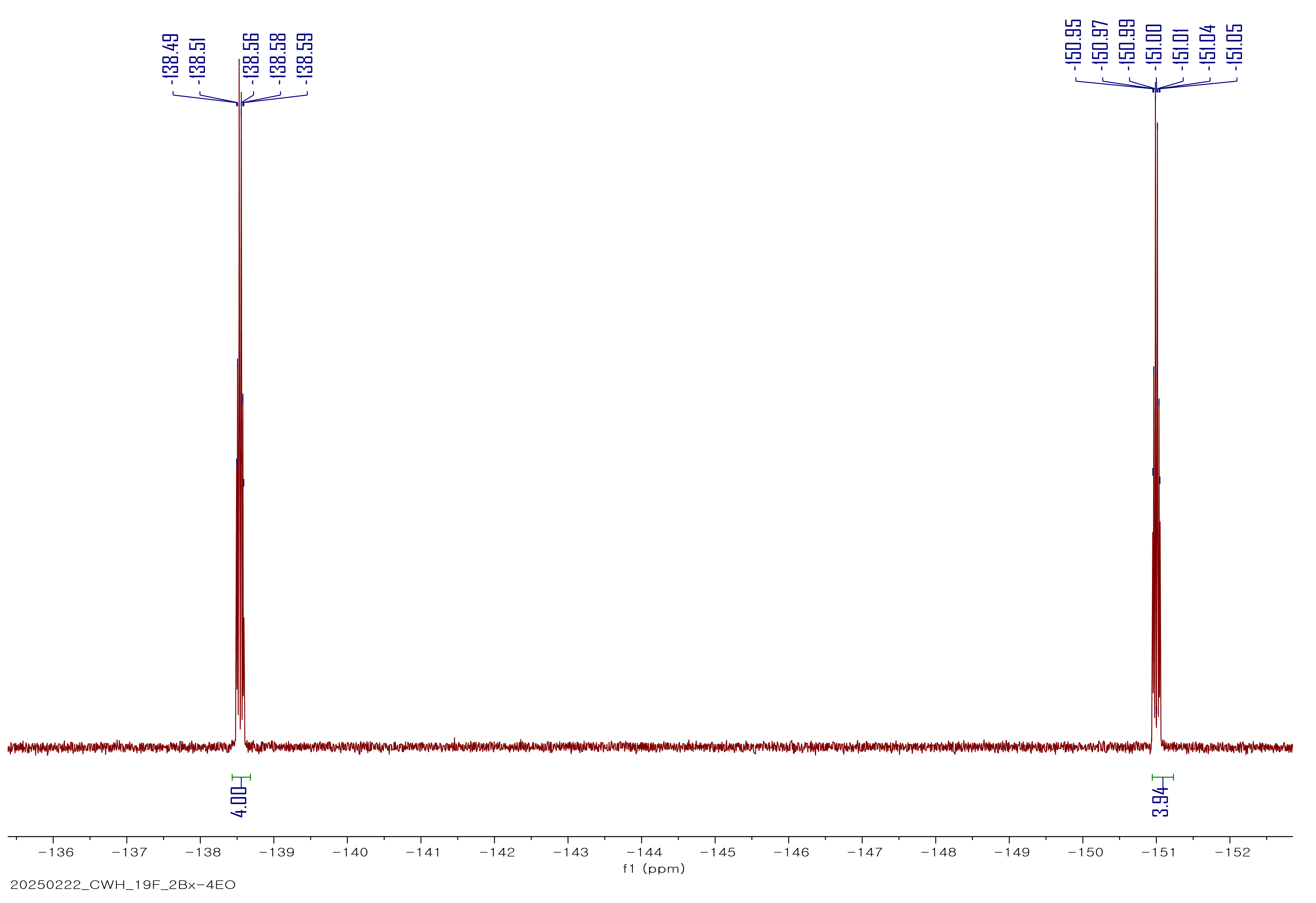


**Figure S2.** ^19^F NMR spectrum of 2Bx-4EO in CDCl_3_.


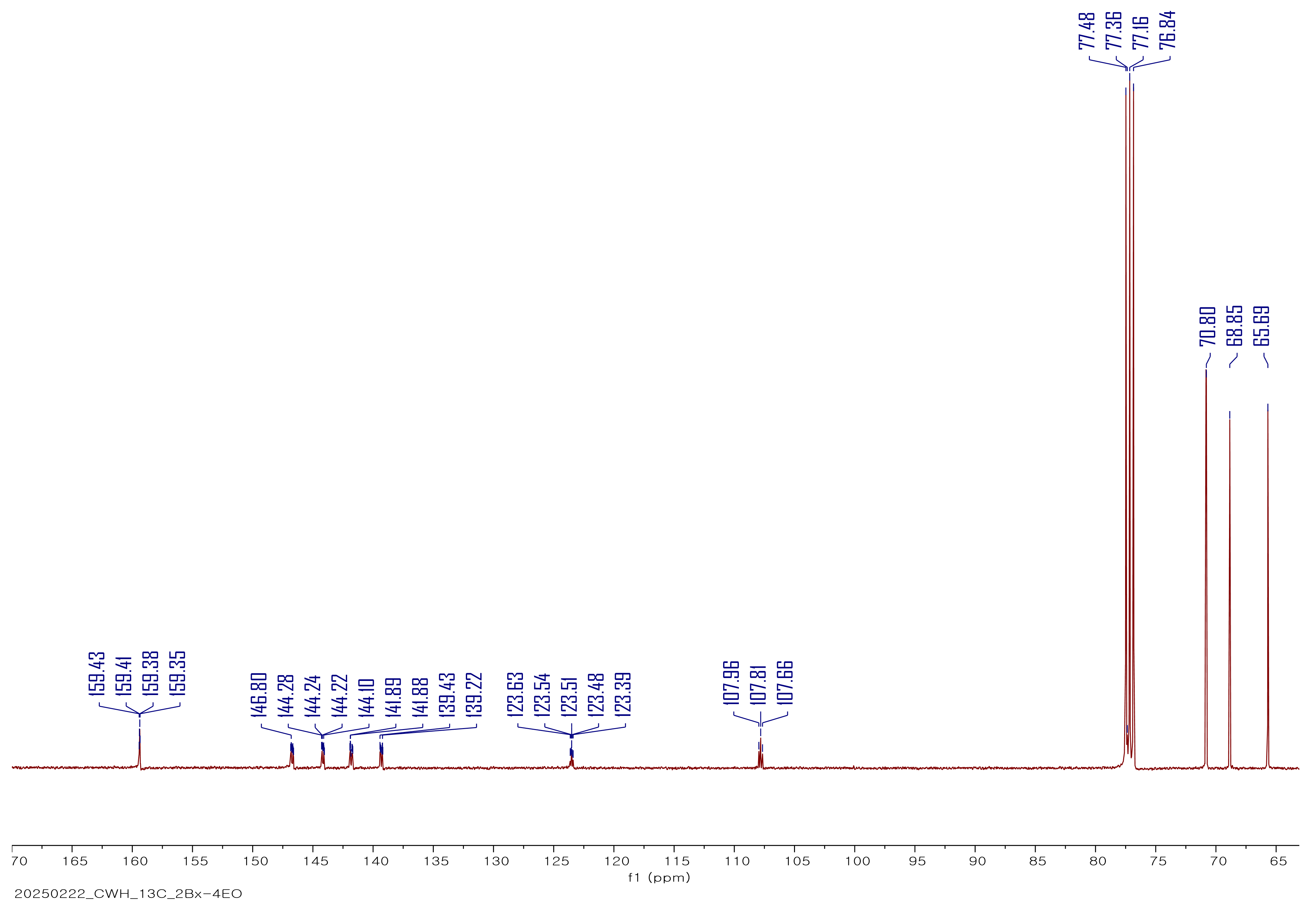


**Figure S3.** ^13^C NMR spectrum of 2Bx-4EO in CDCl_3_.


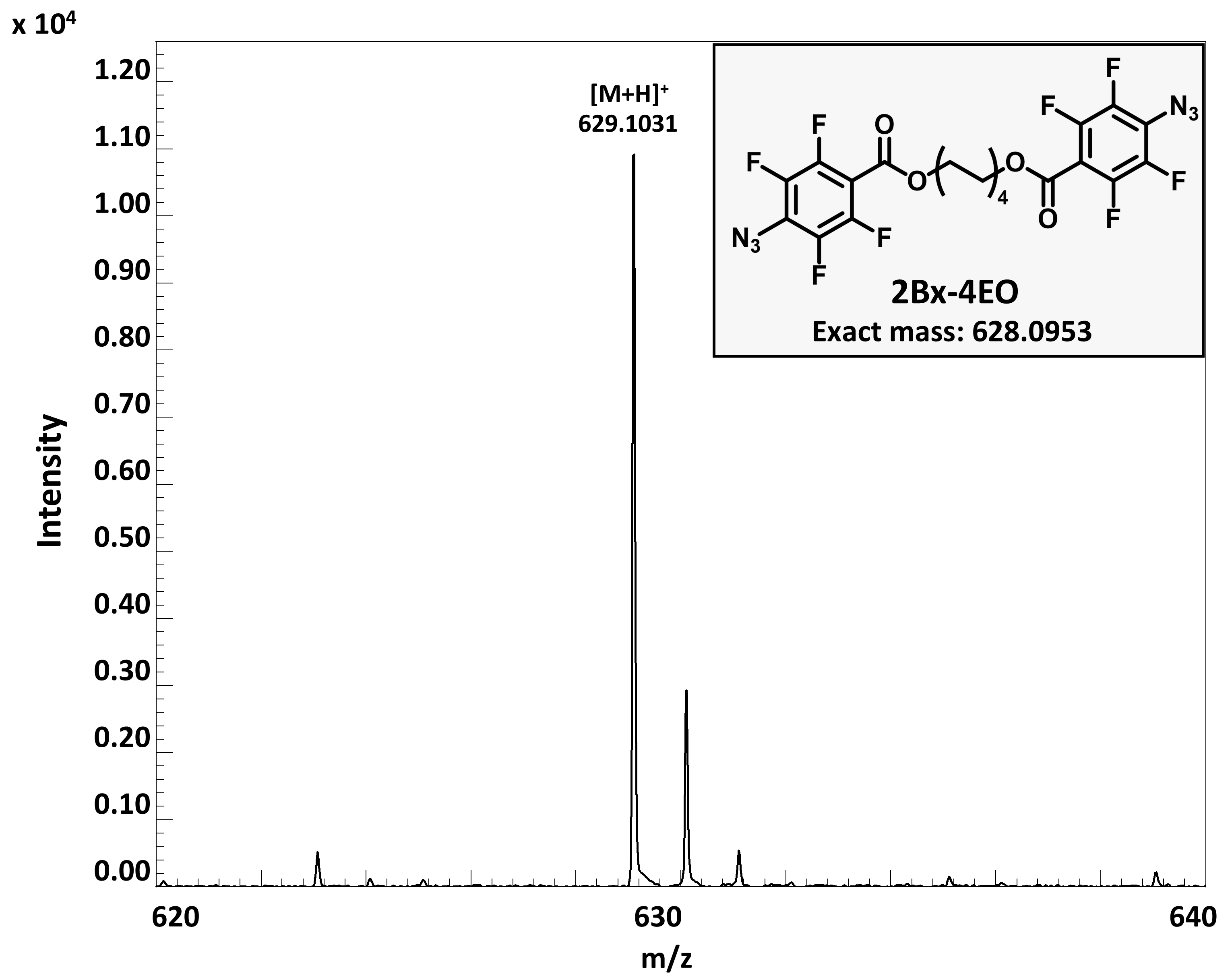


**Figure S4.** ESI-MS spectrum of 2Bx-4EO.


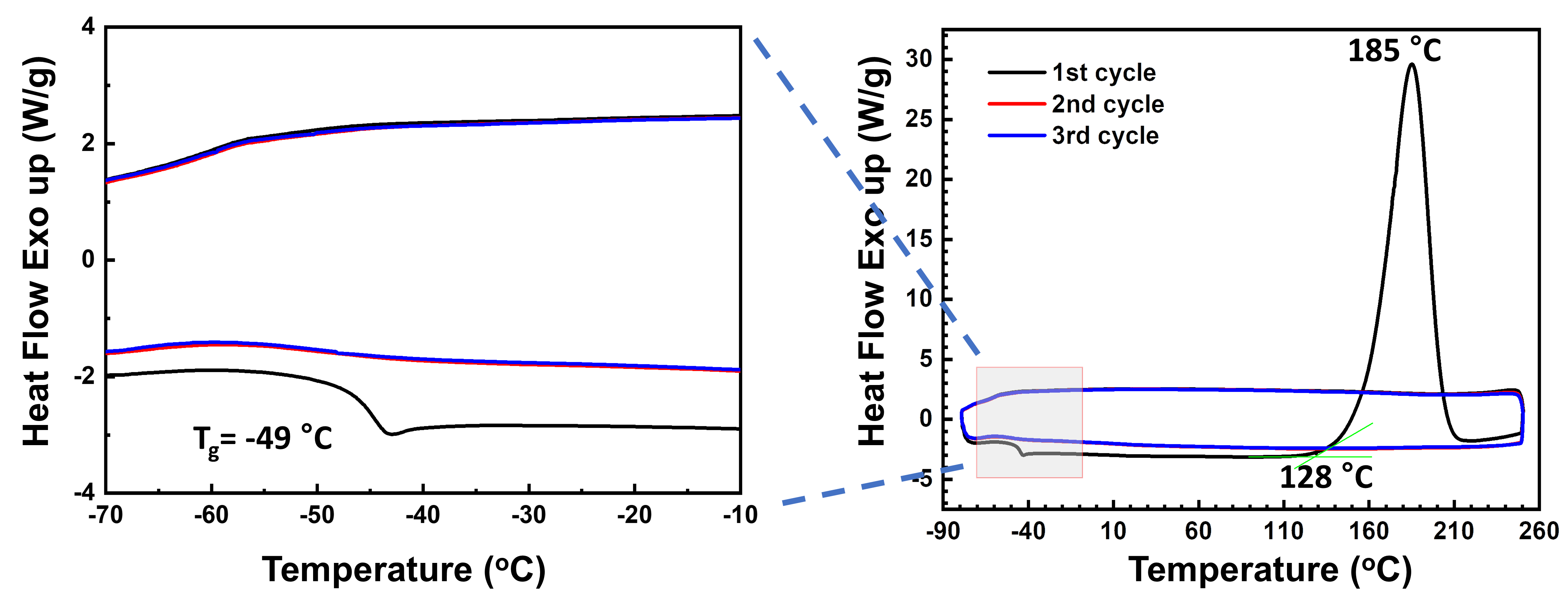


**Figure S5.** DSC measurement of 2Bx-4EO. The glass transition temperature was observed at −49 °C, and the decomposition of the azide group was detected at 128 °C.


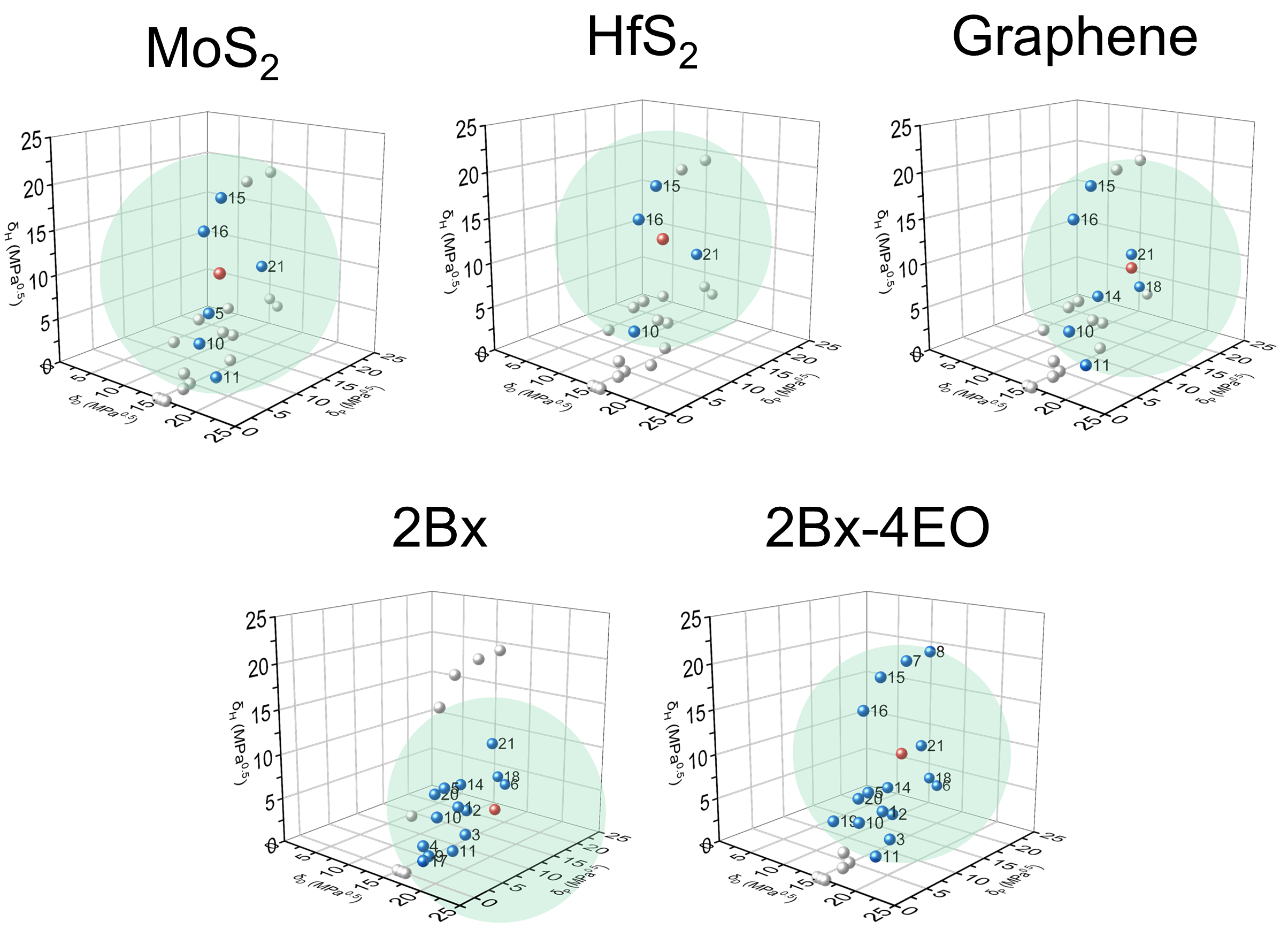


**Figure S6.** Hansen solubility space for MoS_2_, HfS_2_, graphene, 2Bx, and 2Bx-4EO. The blue circles indicate good solvents for each substance, and the gray circles represent poor solvents. The red circle indicates the center of the Hansen solubility sphere.


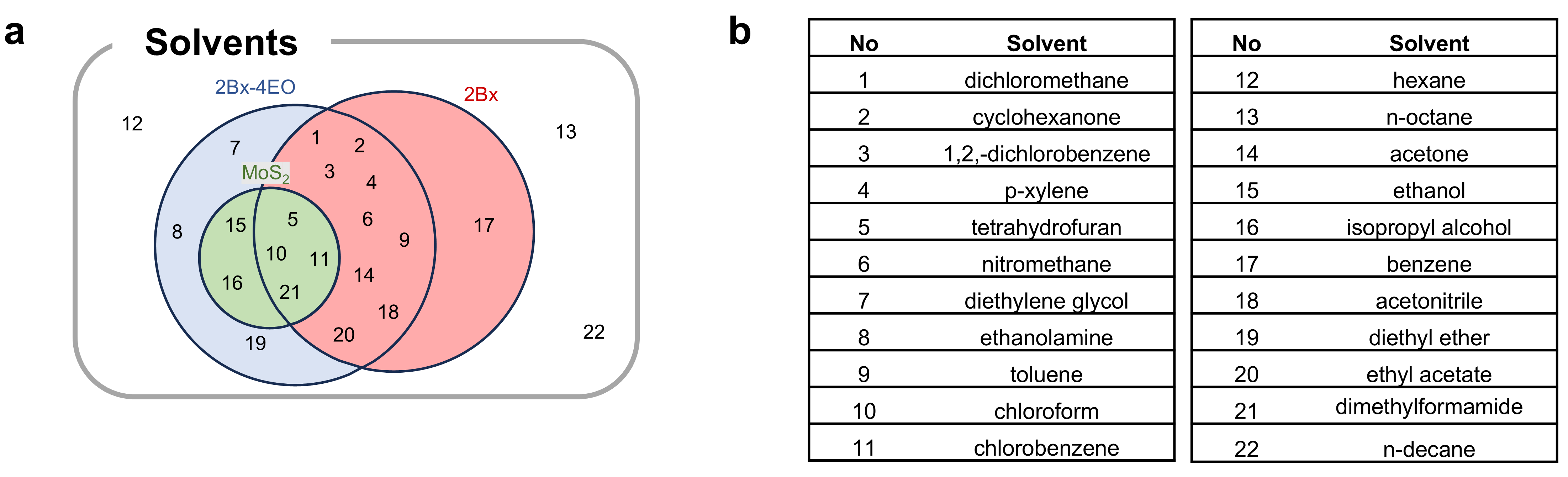


**Figure S7.** a) Venn diagram of the good and poor solvents for 2Bx-4EO (blue), 2Bx (red), and MoS_2_ (green) based on the Hansen solubility parameter analysis results. The intersection indicates common solvents. b) A list of 22 solvent candidates used for the Hansen solubility parameter analysis.


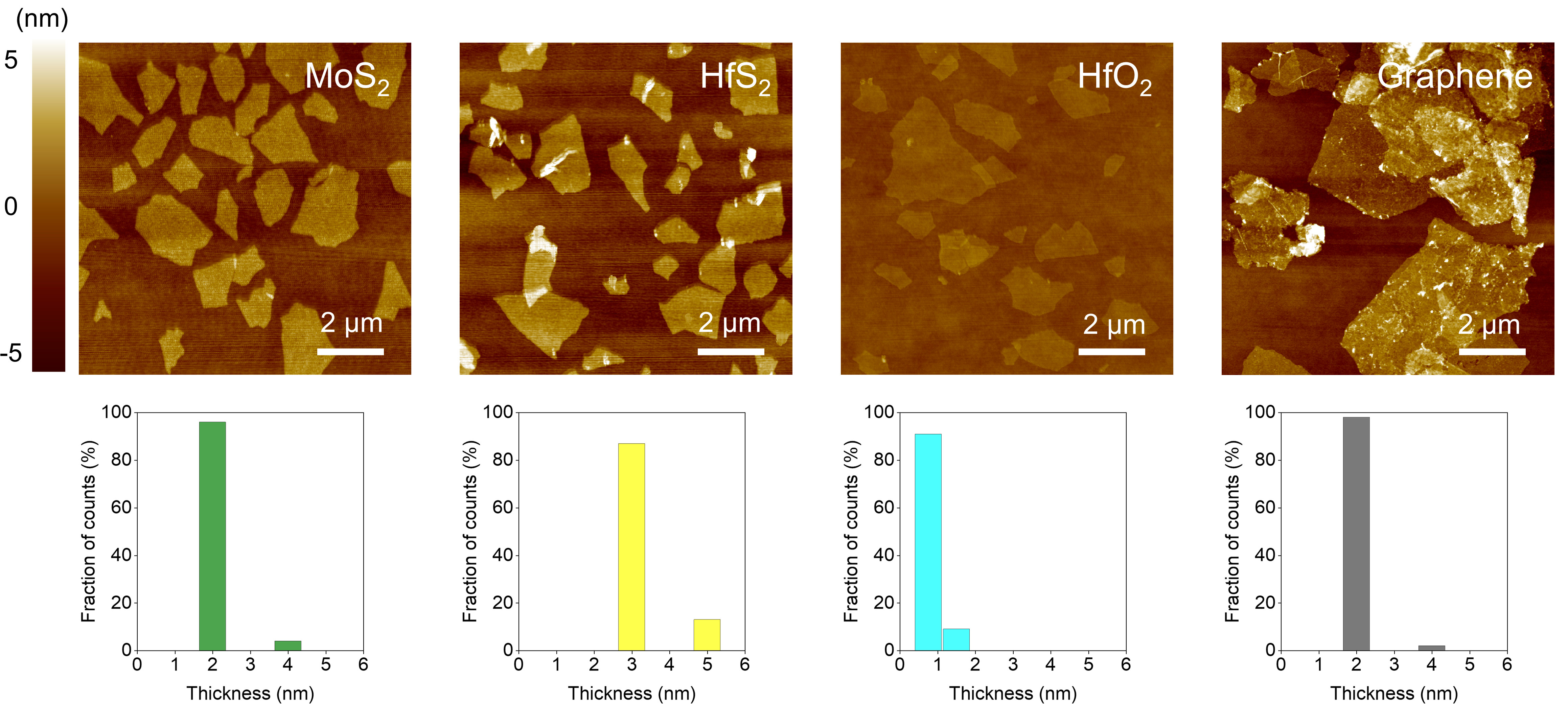


**Figure S8.** AFM images and thickness distributions of the MoS_2_, HfS_2_, HfO_2_ (oxidized from HfS_2_), and graphene nanosheets.


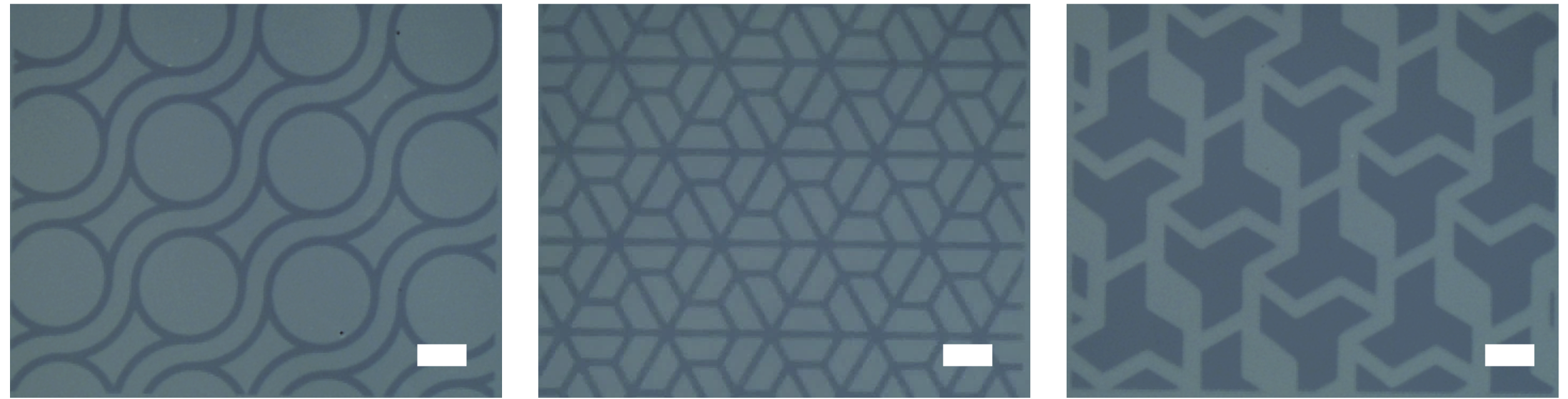


**Figure S9.** Patterned 2D nanoflakes with various shapes. Scale bar: 200 µm.


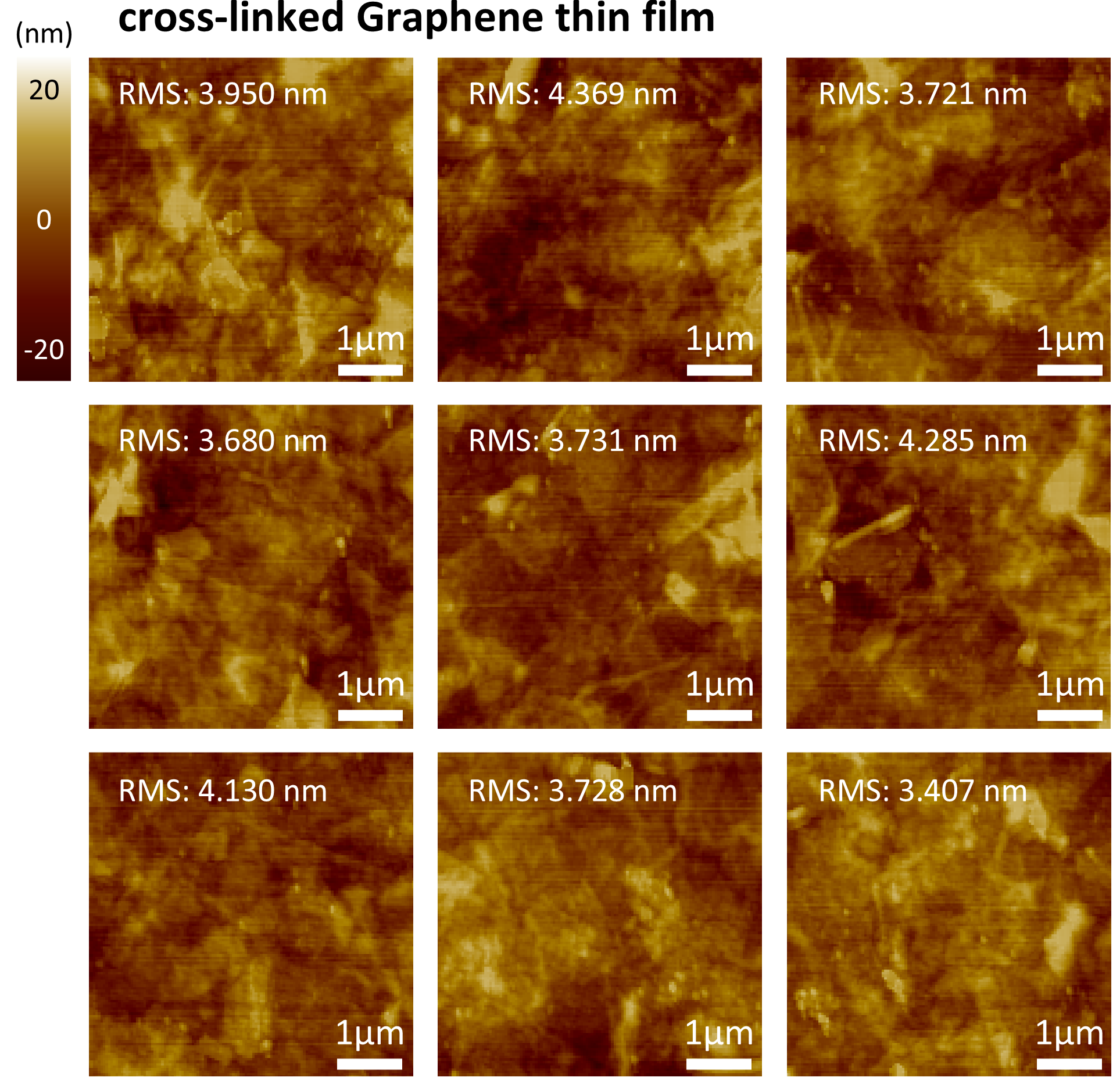


**Figure S10.** AFM images and RMS roughness values of cross-linked graphene thin films.


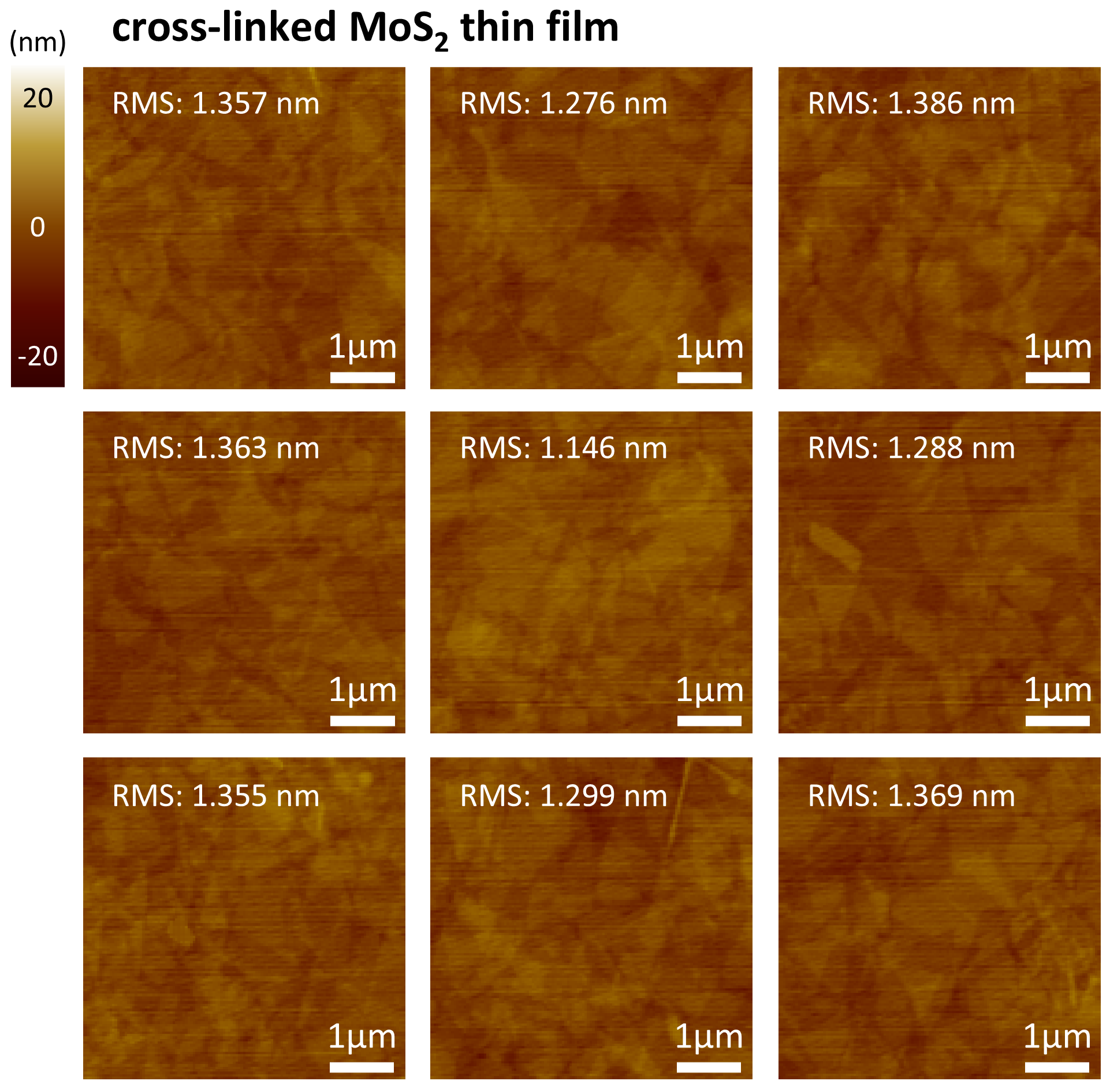


**Figure S11.** AFM images and RMS roughness values of cross-linked MoS_2_ thin films showing minimal RMS variations.


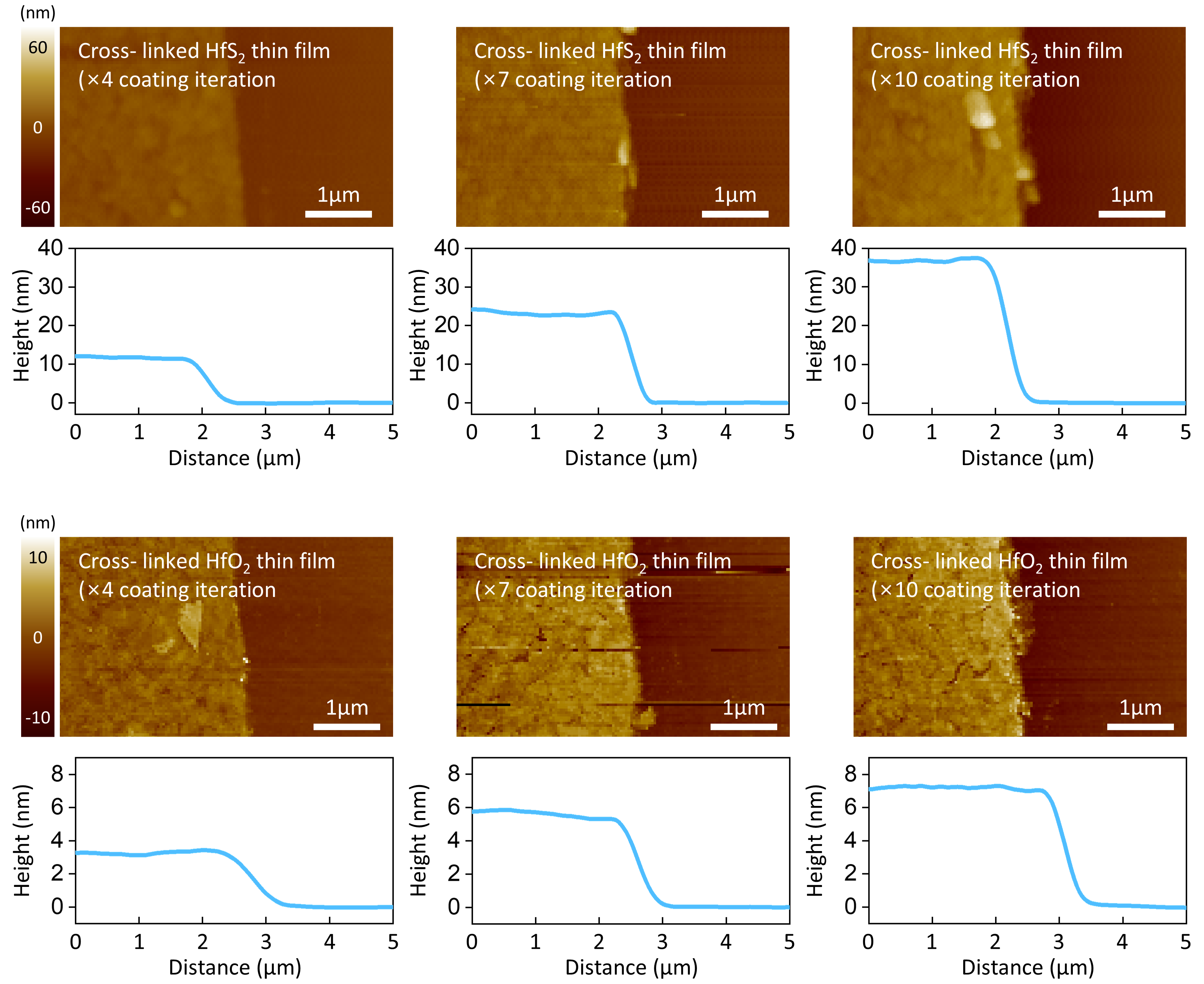


**Figure S12.** AFM images of cross-linked HfS_2_ thin films (top) and cross-linked HfO_2_ (oxidized) thin films (bottom) after multiple coating iterations (4,7, and 10 times), along with their height profiles.


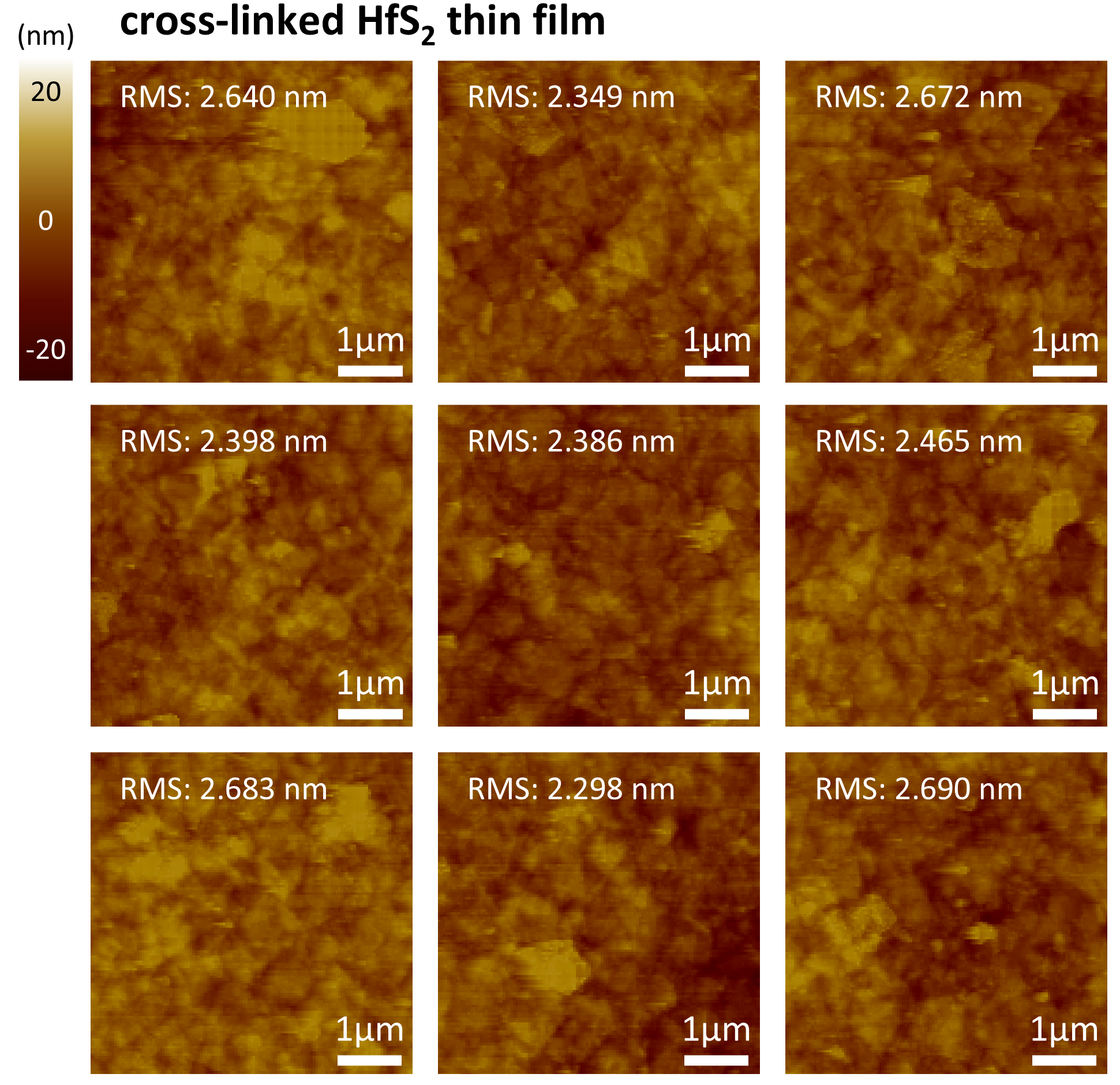


**Figure S13.** AFM images and RMS roughness values of cross-linked HfS_2_ thin films before oxidation.


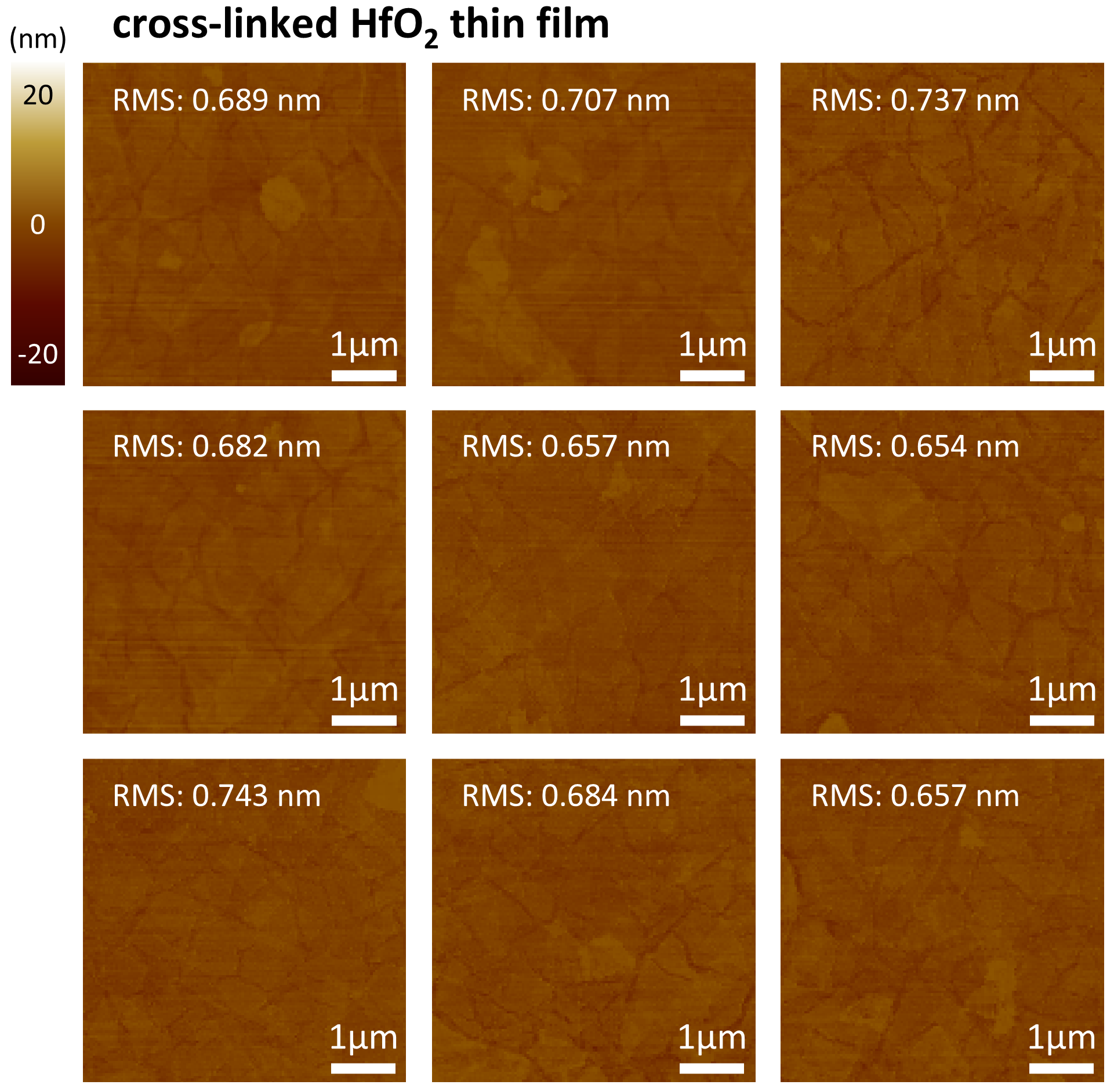


**Figure S14.** AFM images and RMS roughness values of cross-linked HfO_2_ thin films, showing RMS roughness after oxidation.


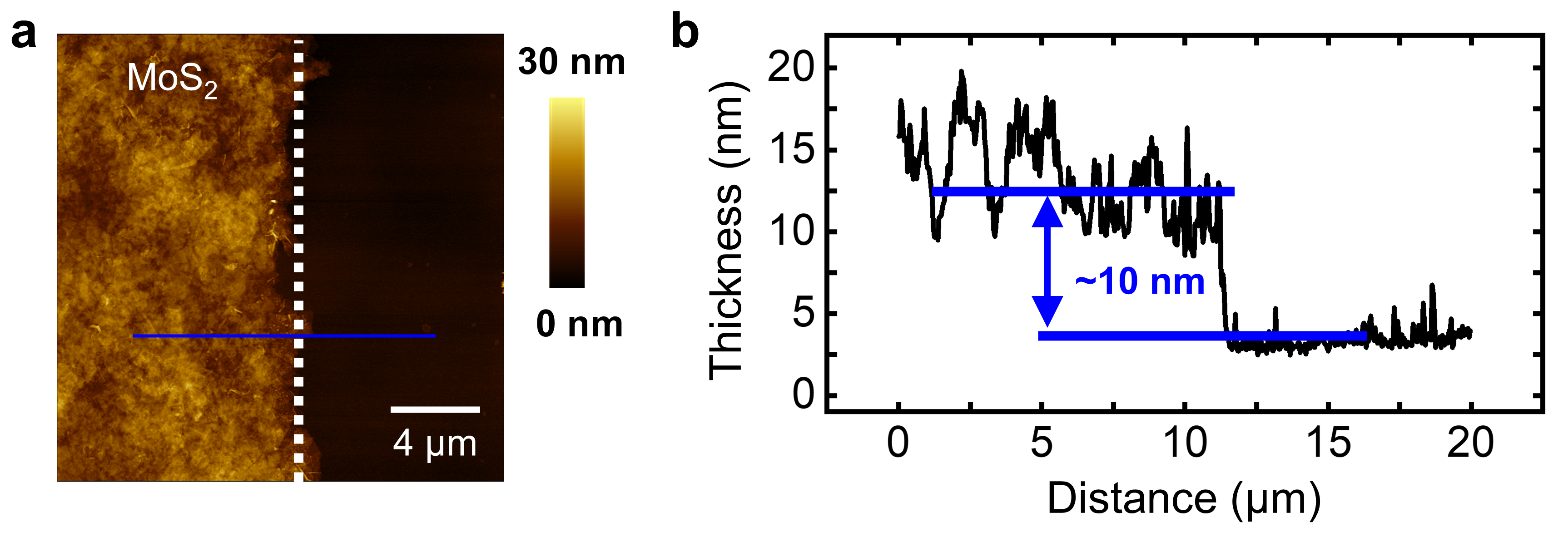


**Figure S15.** **a)** AFM morphology image of the MoS_2_ thin films. **b)** The extracted height profile of MoS_2_ thin films.


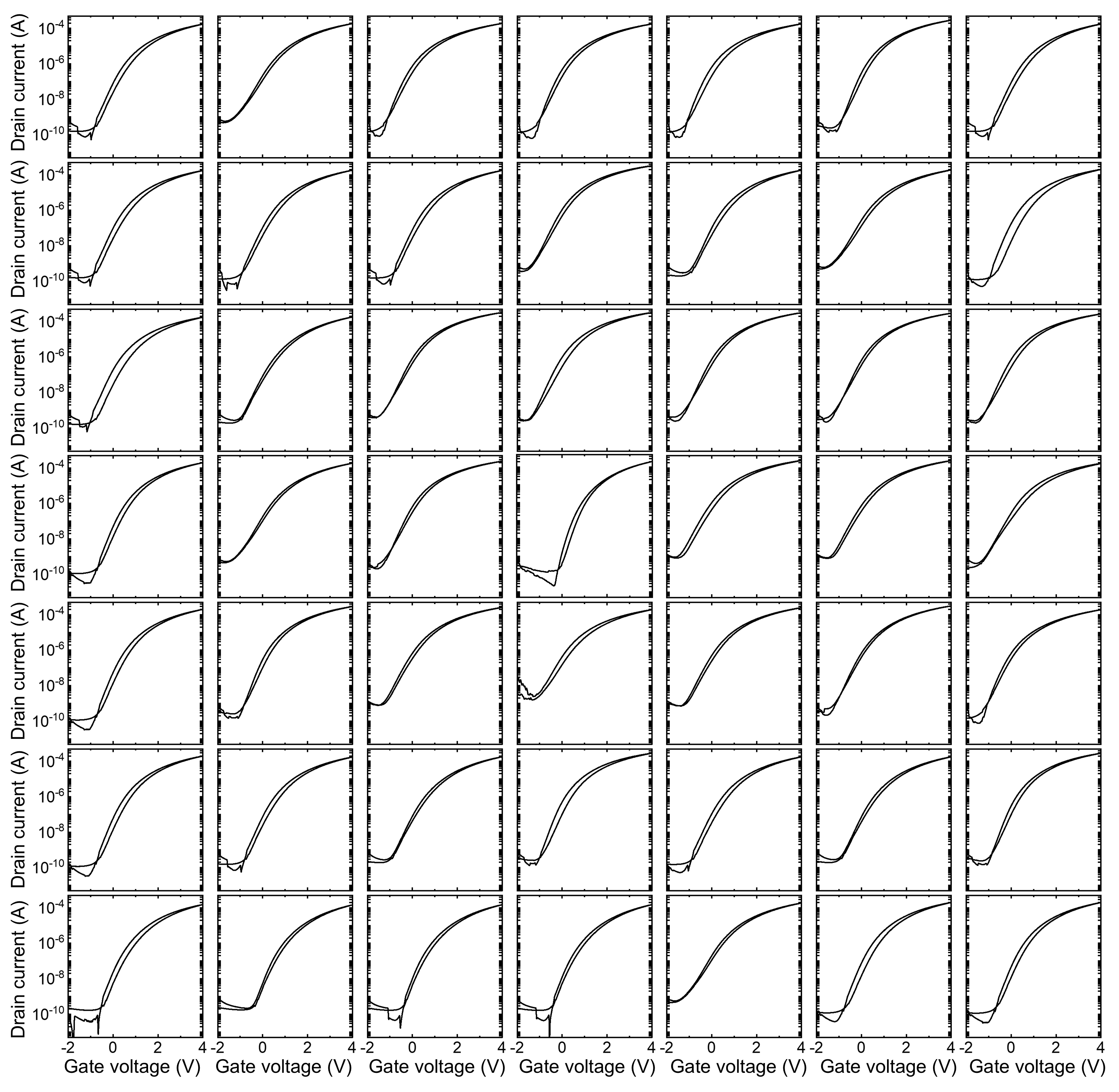


**Figure S16.** Transfer curves of 49 fully photopatterned FET arrays based on cross-linked 2D networks, including semiconducting MoS_2_, conducting graphene, and insulating HfO_2_ oxidized from HfS_2_.


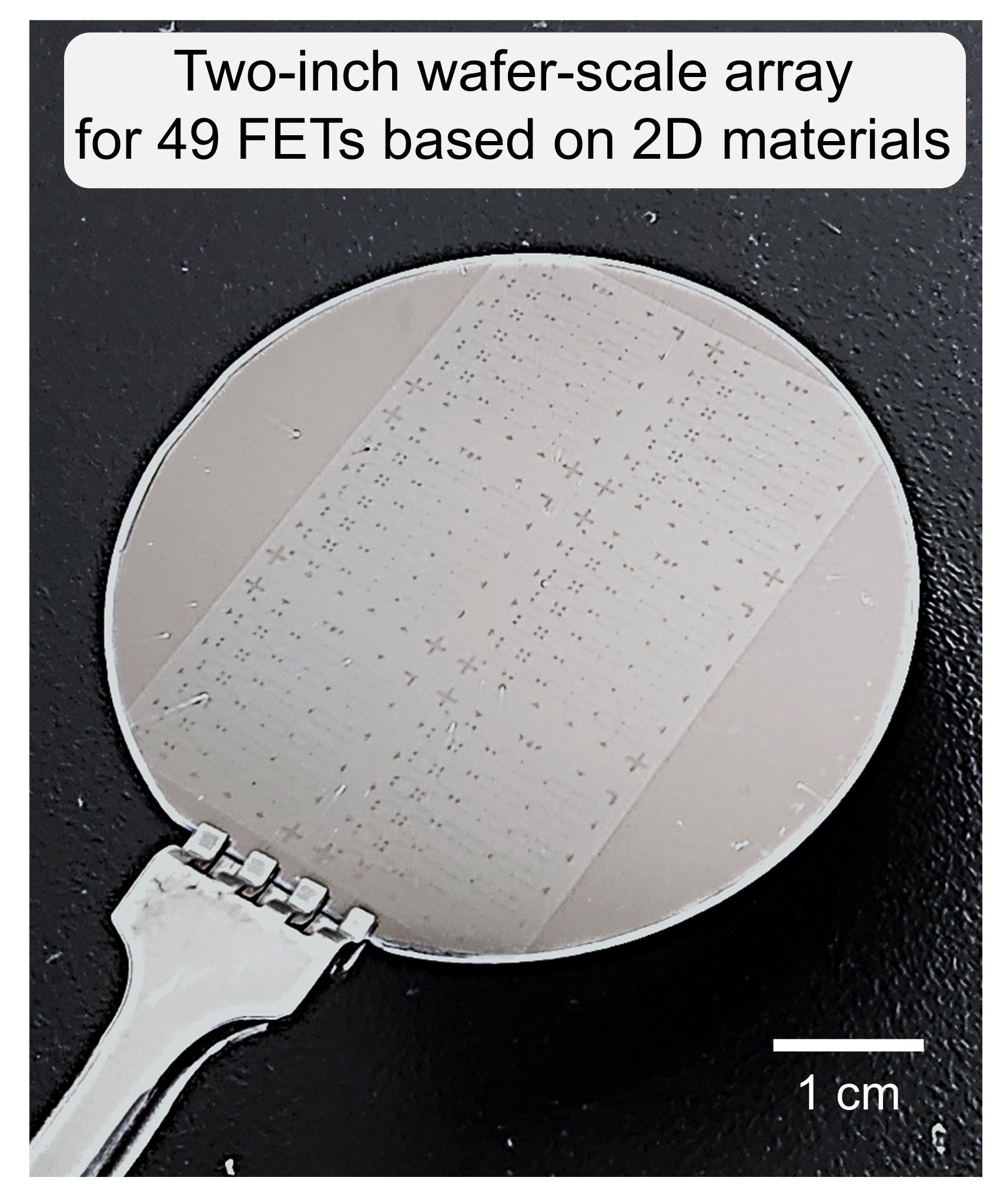


**Figure S17.** Photographic image of a wafer-scale array of the 49 patterned FETs fabricated on a two-inch wafer based on solution-processed 2D materials.


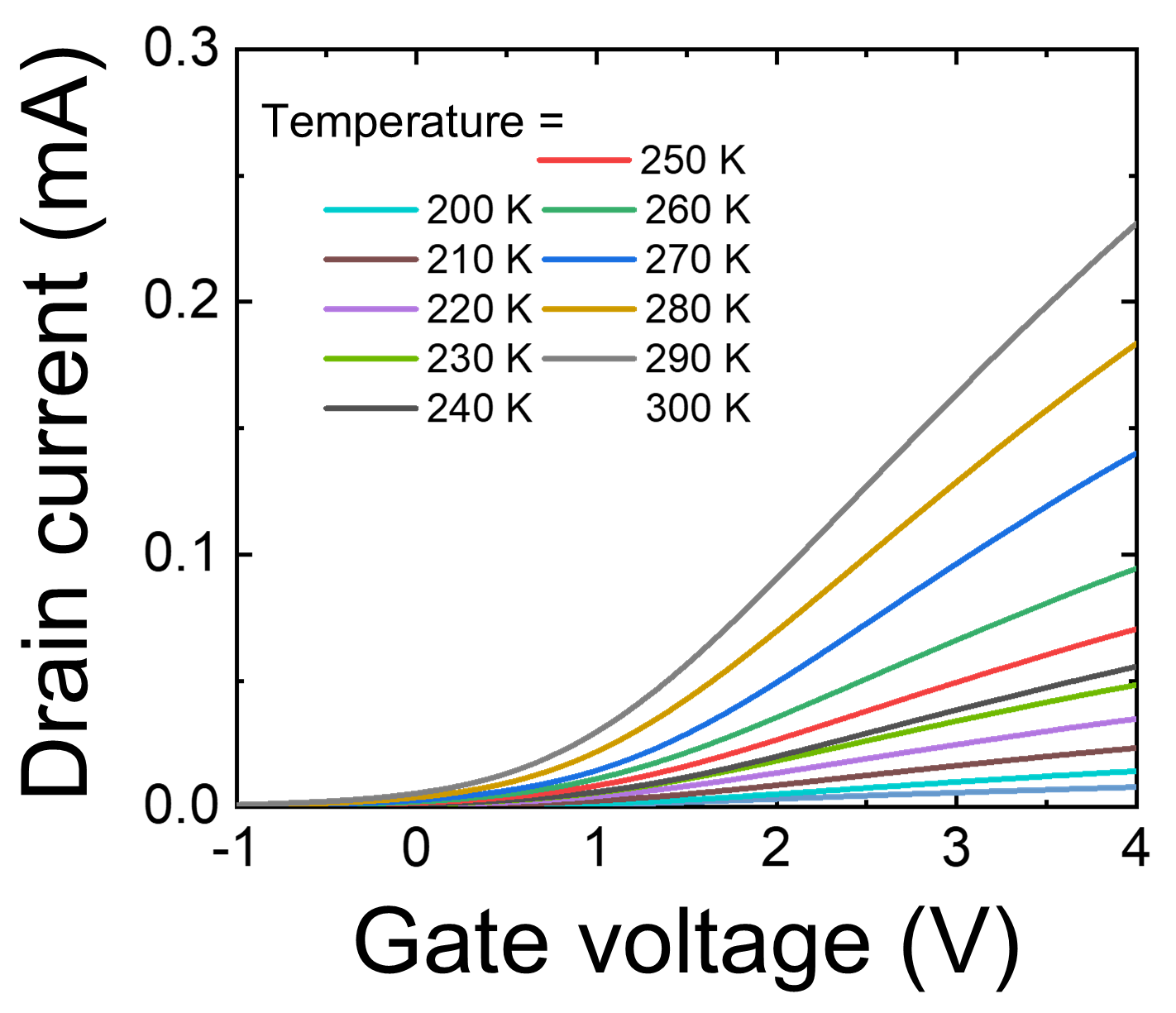


**Figure S18.** Transfer curves of a photopatterned 2D material-based single FET device measured at different temperatures ranging from 200 K to 300 K.


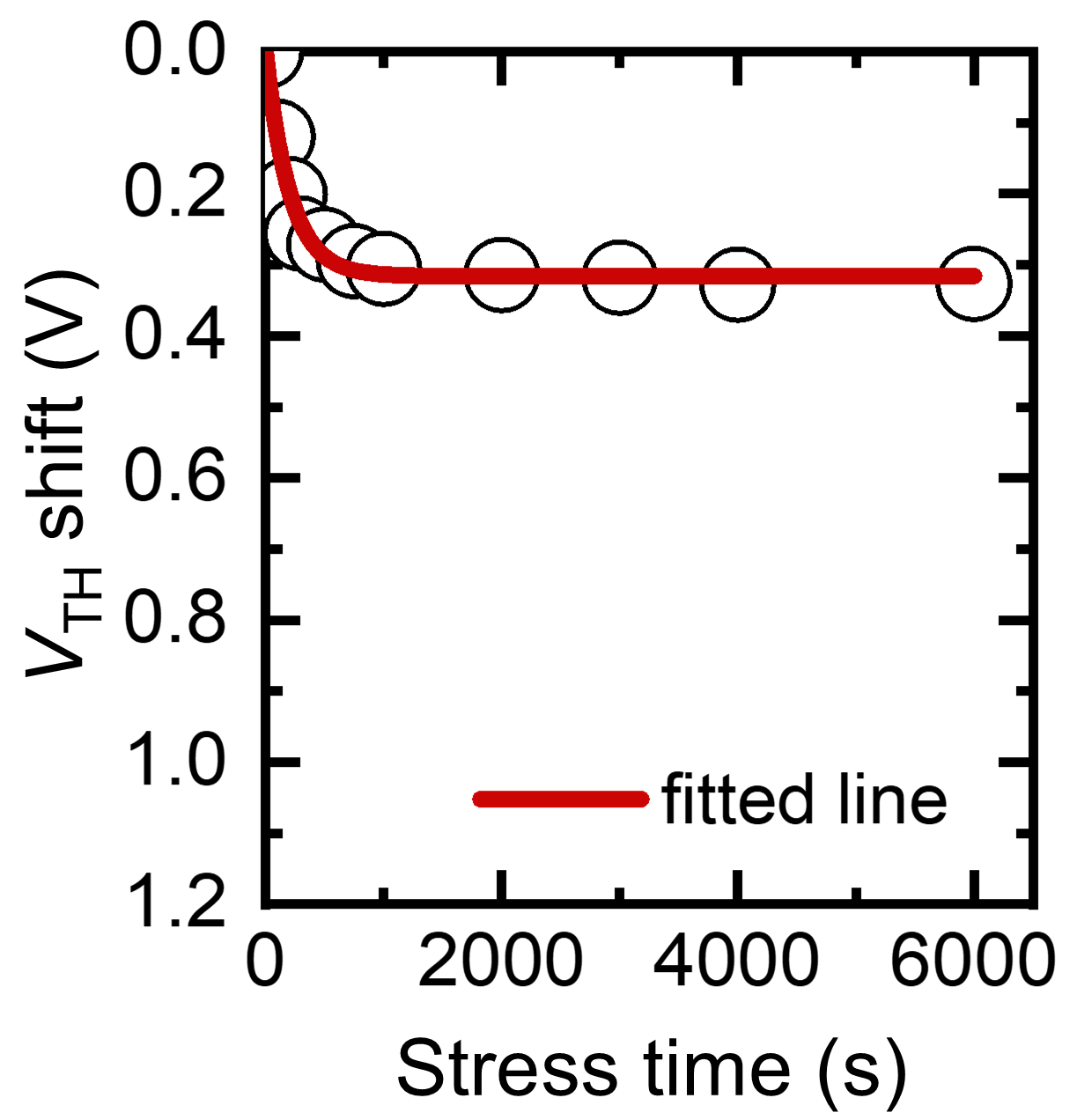


**Figure S19.** Threshold voltage shifts with respect to gate bias stress time.


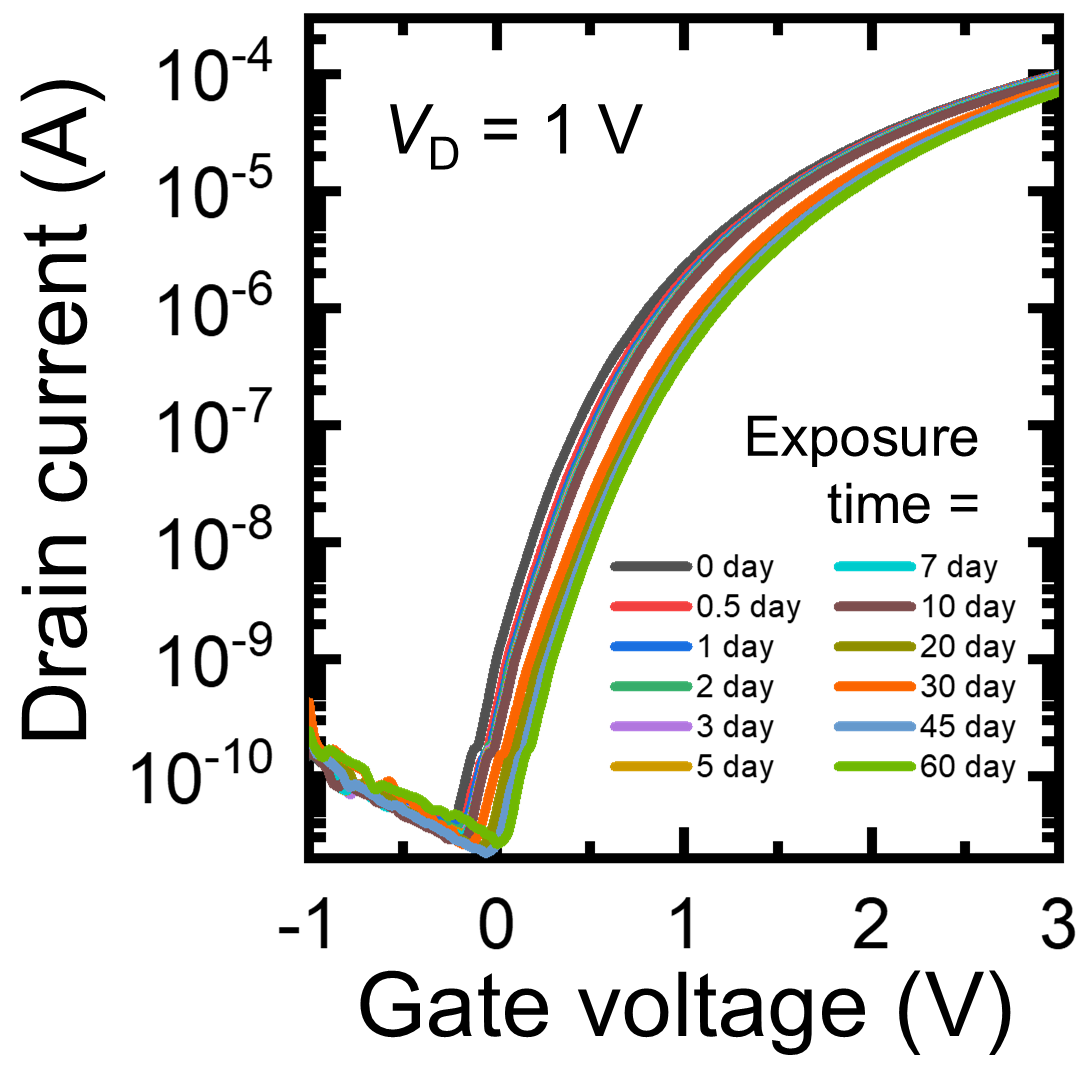


**Figure S20.** A series of transfer curves obtained at different air exposure times under ambient conditions.


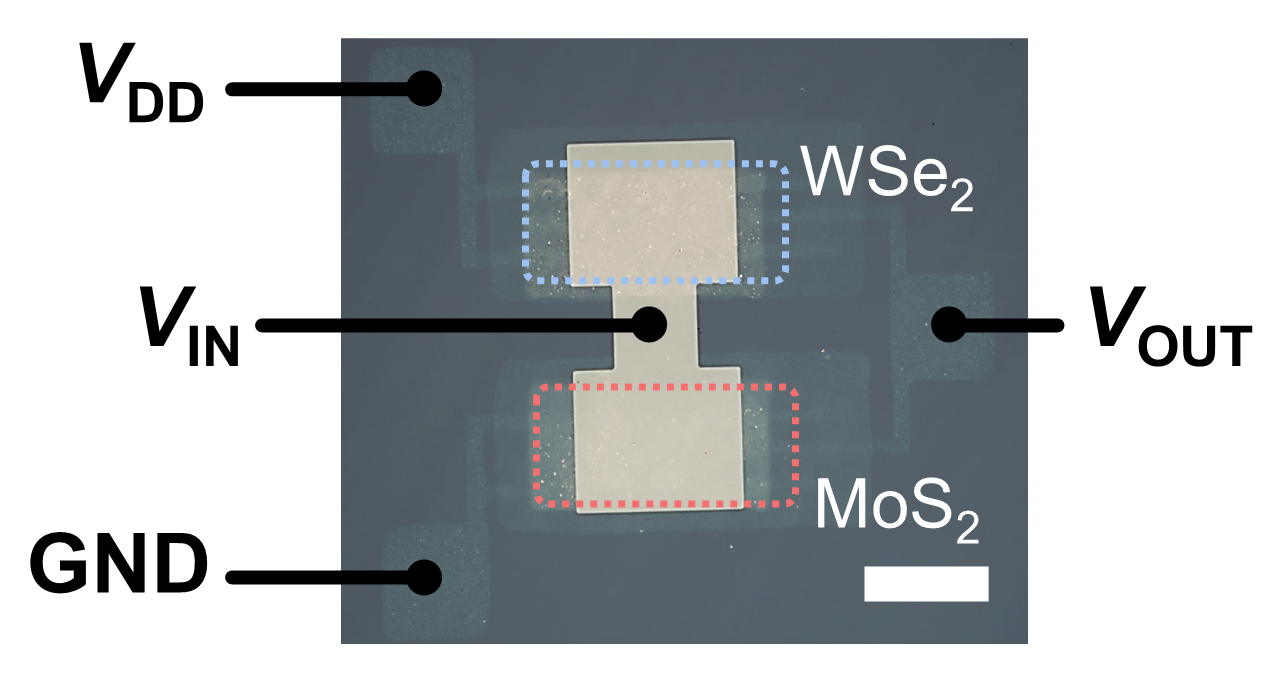


**Figure S21.** Optical microscopic image of the NOT logic gate comprising *p*-type semiconducting WSe_2_ and *n*-type semiconducting MoS_2_ on a single substrate. Scale bar: 400 µm.


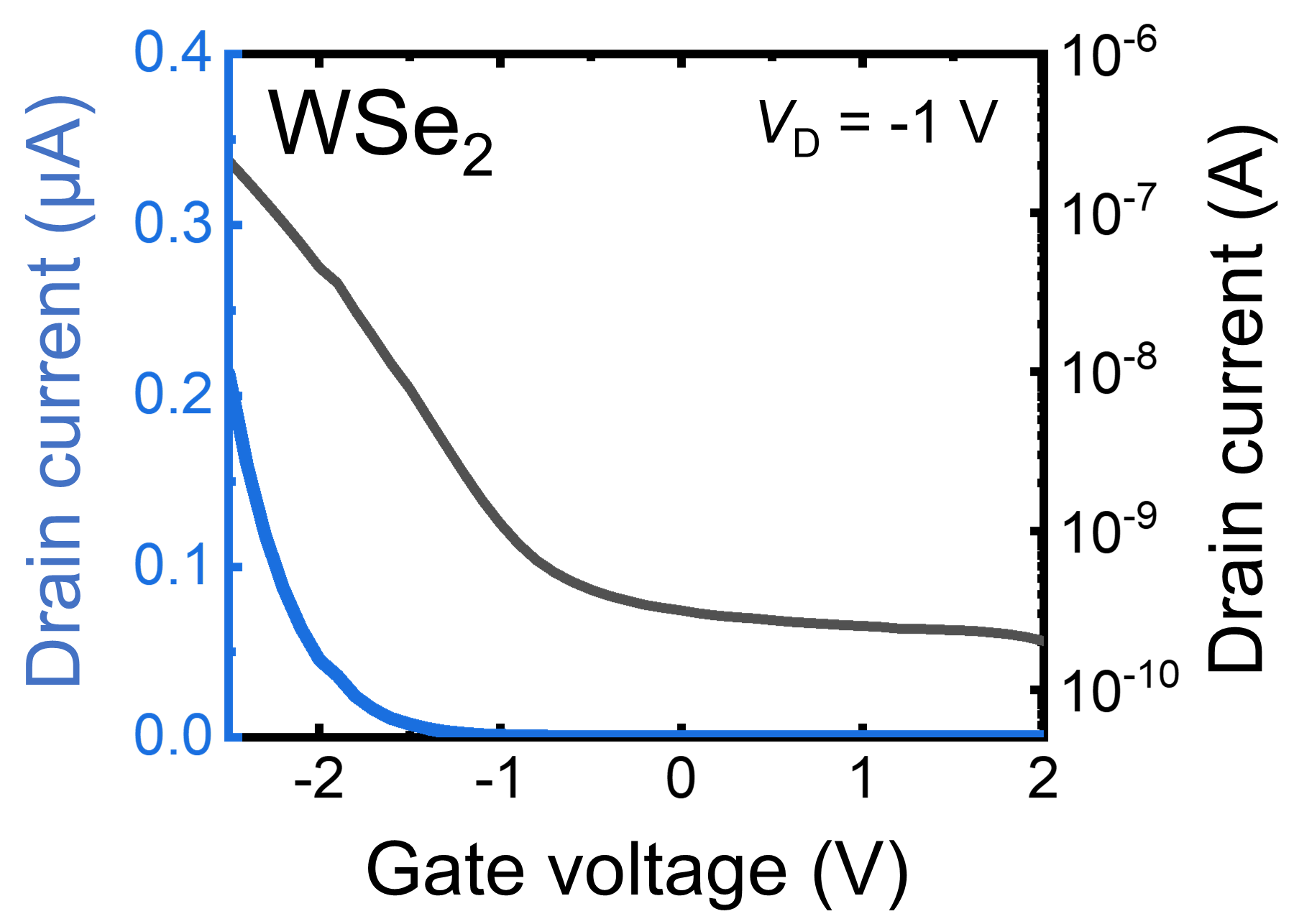


**Figure S22.** Transfer characteristics of a typical *p*-type WSe_2_-based FET.


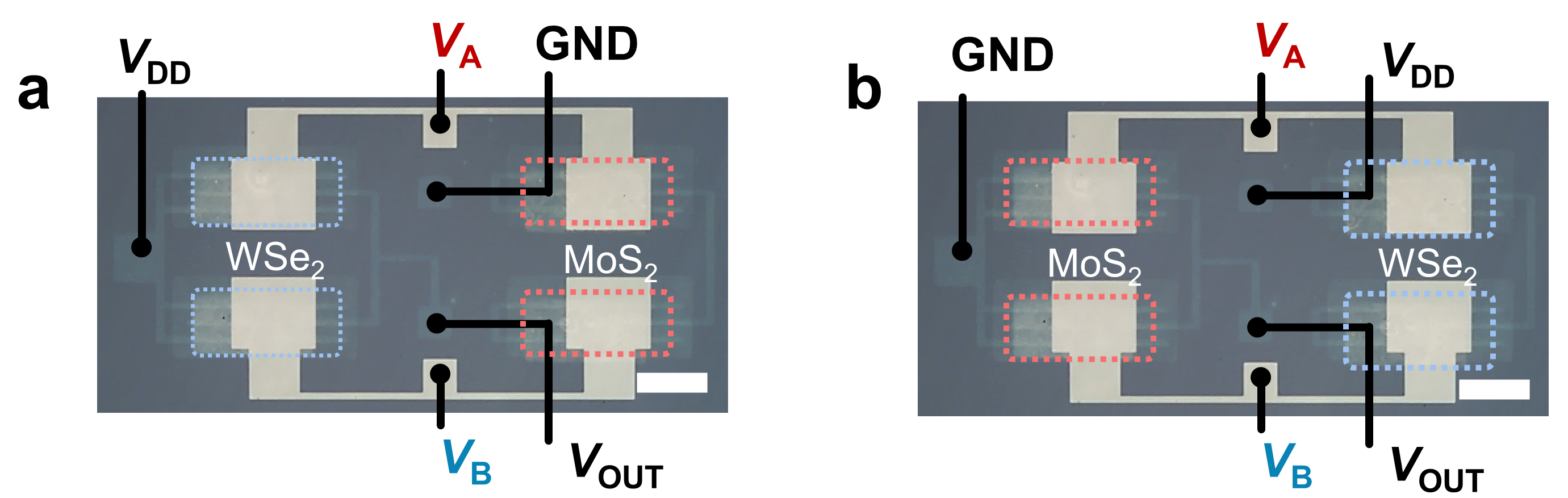


**Figure S23.** Optical microscopic images of a) NAND and b) NOR logic gates comprising graphene, MoS_2_, WSe_2_, and HfO_2_ on a single substrate. Scale bar: 400 µm.

**References.**

1. J. Xue, et al., Solution-processable assembly of 2D semiconductor thin films and superlattices with photoluminescent monolayer inks, *Chem*, **2024**, 10, 1471-1484.

2. S. K. Mondal, et al., Inkjet-printed MoS_2_ transistors with predominantly intraflake transport, *Small methods*, **2021**, 5, 2100634.

3. A. G. Kelly, et al., All-printed thin-film transistors from networks of liquid-exfoliated nanosheets, *Science*, **2017**, 356, 69–73.

4. N. Liu, et al., Large-area atomically thin MoS_2_ nanosheets prepared using electrochemical exfoliation, *ACS Nano*, **2014**, 8, 6902–6910.

5. S. Joung, et al., All-solution-processed high-performance MoS_2_ thin-film transistors with a quasi-2D perovskite oxide dielectric, *ACS Nano*, **2024**, 18, 1958–1698.

6. X. Sui, et al., Fully inkjet-printed, 2D materials-based field-effect transistor for water sensing, *Adv. Mater. Technol.*, **2023**, 8, 2301288.

7. M. Chen, et al., Inkjet-printed MoS_2_ nanoplates on flexible substrates for high-performance field effect transistors and gas sensing applications, *ACS Appl. Nano Mater.*, **2023**, 6, 3236–3244.

8. J. Li, et al., Inkjet printing of MoS_2_, *Adv. Funct. Mater.*, **2014**, 24, 6524–6531.

9. L. Li, et al., Interface capture effect printing atomic-thick 2D semiconductor thin films, *Adv. Mater.*, **2022**, 34, 2207392.

10. J. Kim, et al., Area-selective chemical doping on solution-processed MoS_2_ thin-film for multi-valued logic gates, *Nano Lett.*, **2022**, 22, 570–577.

11. C. Ma, et al., Two-dimensional van der Waals thin film transistors as active matrix for spatially resolved pressure sensing, *Nano Res.*, **2021**, 14, 3395–3401.

12. T. Carey, et al., High-mobility flexible transistors with low-temperature solution-processed tungsten dichalcogenides, *ACS Nano*, **2023**, 17, 2912–2922.

13. T. Zou, et al., High-performance solution-processed 2D *p*-type WSe_2_ transisotrs and circuits through molecular doping, *Adv. Mater.*, **2023**, 35, 2208934.
